# Extreme Environmental Variability Induces Frontloading of Coral Biomineralisation Genes to Maintain Calcification Under pCO_2_ Variability

**DOI:** 10.1111/mec.17603

**Published:** 2024-11-28

**Authors:** Kristen T. Brown, Zoe Dellaert, Marcelina P. Martynek, Julia Durian, Tali Mass, Hollie M. Putnam, Katie L. Barott

**Affiliations:** ^1^ Department of Biology University of Pennsylvania Philadelphia Pennsylvania USA; ^2^ School of Biological Sciences University of Queensland St. Lucia Queensland Australia; ^3^ Department of Biological Sciences University of Rhode Island Kingston Rhode Island USA; ^4^ Oberlin College Oberlin Ohio USA; ^5^ Department of Marine Biology, Leon H. Charney School of Marine Sciences University of Haifa Haifa Israel

**Keywords:** biomineralisation, calcification, coral reefs, environmental variability, extreme environments, local adaptation, ocean acidification, priming

## Abstract

Corals residing in habitats that experience high‐frequency seawater pCO_2_ variability may possess an enhanced capacity to cope with ocean acidification, yet we lack a clear understanding of the molecular toolkit enabling acclimatisation to environmental extremes or how life‐long exposure to pCO_2_ variability influences biomineralisation. Here, we examined the gene expression responses and micro‐skeletal characteristics of *Pocillopora damicornis* originating from the reef flat and reef slope of Heron Island, southern Great Barrier Reef. The reef flat and reef slope had similar mean seawater pCO_2_, but the reef flat experienced twice the mean daily pCO_2_ amplitude (range of 797 v. 399 μatm day^−1^, respectively). A controlled mesocosm experiment was conducted over 8 weeks, exposing *P. damicornis* from the reef slope and reef flat to stable (218 ± 9) or variable (911 ± 31) diel pCO_2_ fluctuations (μatm; mean ± SE). At the end of the exposure, *P. damicornis* originating from the reef flat demonstrated frontloading of 25% of the expressed genes regardless of treatment conditions, suggesting constitutive upregulation. This included higher expression of critical biomineralisation‐related genes such as carbonic anhydrases, skeletal organic matrix proteins, and bicarbonate transporters. The observed frontloading corresponded with a 40% increase of the fastest deposited areas of the skeleton in reef flat corals grown under non‐native, stable pCO_2_ conditions compared to reef slope conspecifics, suggesting a compensatory response that stems from acclimatisation to environmental extremes and/or relief from stressful pCO_2_ fluctuations. Under escalating ocean warming and acidification, corals acclimated to environmental variability warrant focused investigation and represent ideal candidates for active interventions to build reef resilience while societies adopt strict policies to limit climate change.

## 1 Introduction

Unmitigated carbon dioxide (CO_2_) emissions are causing the acidification of our oceans through increasing seawater pCO_2_, which decreases oceanic pH (Feely, Doney, and Cooley 2009; Hoegh‐Guldberg et al. 2007). Coral reefs are amongst the marine ecosystems most threatened by ocean acidification, as increasing pCO_2_ leads to the weakening and dissolution of the calcium carbonate (CaCO_3_) frameworks that form the foundation of the reef structure (Dove et al. 2020; Eyre et al. 2018). Coral reefs are the largest biogenic structures on Earth and are formed primarily via the biomineralisation activity of reef‐building corals. The collapse of these structures will have a multitude of adverse impacts, including: (i) reductions in the dissipation of wave energy that prevent coastal inundation from storm surges and sea‐level rise (Harris et al. 2018); (ii) the degradation of three‐dimensional habitat that supports ecologically and economically important fisheries (Rogers, Blanchard, and Mumby 2014); and (iii) increases in coastal erosion of tropical reef islands and beaches (Ferrario et al. 2014). As climate change intensifies and sea levels continue to rise, it has never been more important to identify acidification‐resilient corals that are capable of building and maintaining these critical reef structures in the Anthropocene.

Reef‐building corals thrive across many distinct reef geomorphological habitats, including those that expose inhabitants to a high frequency of short‐term temperature and pCO_2_ extremes (Brown et al. 2022; Camp et al. 2019). While exposure to temperature variability has been widely recognised as a mechanism that can promote elevated thermal tolerance (Barshis et al. 2013; Brown, Martynek, and Barott 2024; Kenkel and Matz 2016; Safaie et al. 2018; Voolstra et al. 2020), the role of seawater pH variability as a mechanism to promote coral acidification resilience is poorly understood (Rivest, Comeau, and Cornwall 2017). Tolerance to environmental variability likely stems from adaptation and/or acclimatisation, the latter of which includes mechanisms such as phenotypic plasticity, harbouring stress‐tolerant symbiont communities, higher baseline expression of stress response genes, and/or epigenetics (Bay and Palumbi 2014; Palumbi et al. 2014; Kenkel and Matz 2016; Putnam 2021). Interestingly, several studies have identified that natural acidification analogs may prime corals to cope with acidification stress through the enhanced capacity to regulate acid–base homeostasis (Brown et al. 2022; Kenkel et al. 2018; Scucchia, Malik, Putnam, et al. 2021). The influence of seawater pCO_2_ variability on coral biomineralisation is, however, less clear and has been hindered by the inability to disentangle the effects of co‐occurring physical conditions that exist across extreme environments. For example, pCO_2_ variability often varies with wave exposure, such that lagoons that experience extreme pCO_2_ oscillations are also protected from strong wave action and currents, thus patterns in growth may be the product of a more protected location (Brown et al. 2022; Scucchia et al. 2023). However, even in studies that have seemingly isolated seawater pCO_2_ fluctuations, contrasting responses have been observed where oscillatory pH regimes can promote calcification in some species (Comeau et al. 2014) but not others (Cornwall et al. 2018). As such, whether long‐term exposure to seawater pCO_2_ variability results in acidification resilience remains unresolved, yet critical to predicting coral reef growth and persistence in a changing ocean.

**FIGURE 1:**
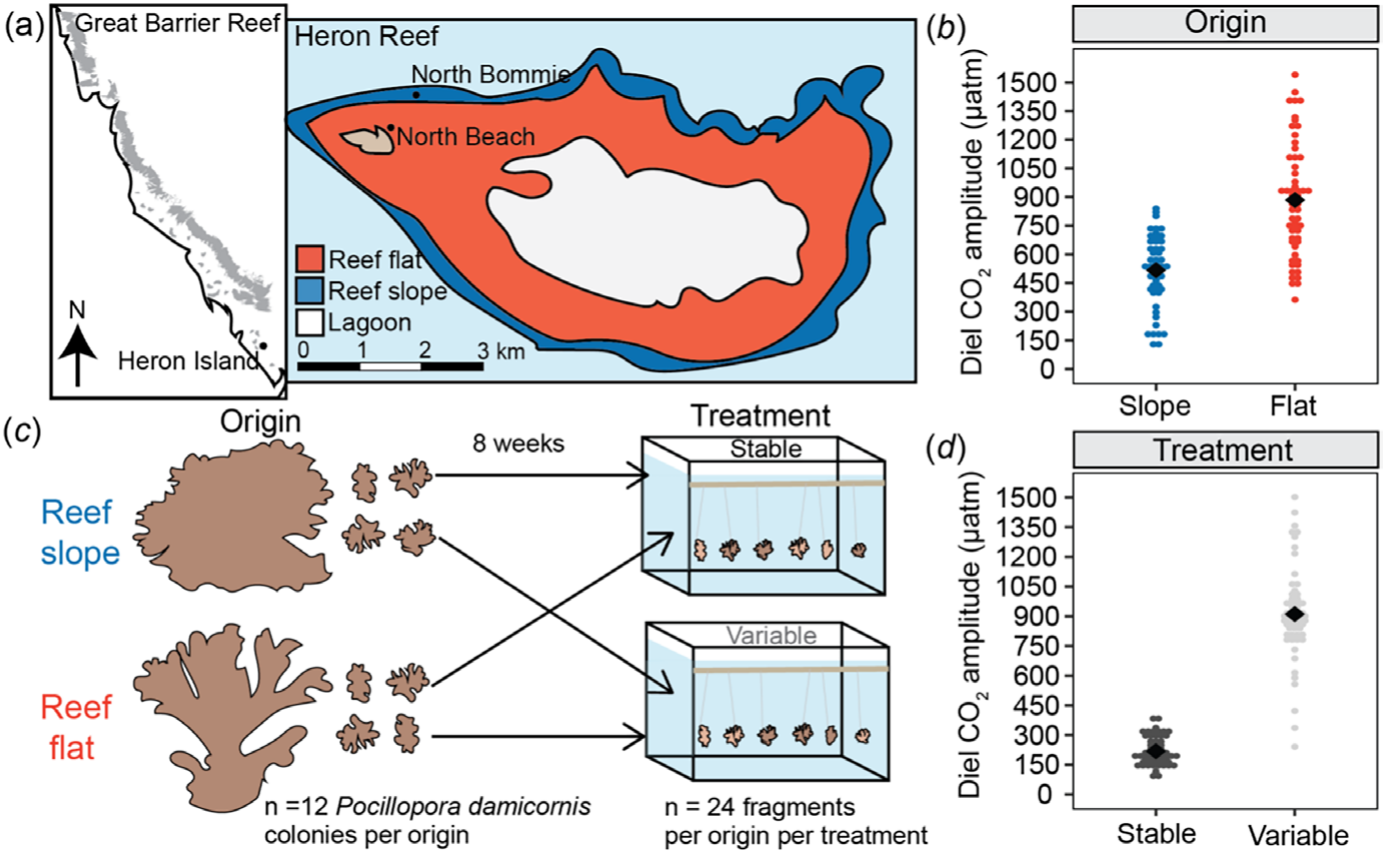
Experimental design and treatment conditions. (a) Cartoon map displaying the geomorphological zones and study sites at Heron Island. (b) Daily seawater pCO_2_ amplitude by origin were recorded in situ within the reef slope and reef flat in 2016; black diamonds indicate the mean. (c) Experimental design, where corals were collected from the reef slope and reef flat and reciprocally transplanted into respective treatment conditions. (d) Daily seawater pCO_2_ amplitude by treatment measured within the experimental tanks; black diamonds indicate the mean. Adapted from (Brown et al. 2022).

To better understand the acclimatisation potential of reef‐building corals to ocean acidification, we assessed the influence of diel seawater pCO_2_ variability on the molecular, physiological, and morphological responses of the coral *Pocillopora damicornis* from two reef locations with contrasting physicochemical conditions. This study focused on the lagoonal reef flat and oceanic reef slope of Heron Island, southern Great Barrier Reef, where tidally‐driven fluctuations in seawater temperature and pCO_2_ result in greater daily environmental variability on the reef flat (up to 4.3°C day^−1^ and 797 μatm day^−1^) compared to the reef slope (up to 1.3°C day^−1^ and 399 μatm day^−1^; Figure 1; Brown et al. 2022). A controlled laboratory study was conducted in aquaria over 8 weeks, reciprocally exposing *P. damicornis* from the reef slope and reef flat to stable or variable diel pCO_2_ fluctuations mimicking those measured on each reef habitat. This allowed us to assess the effect of pCO_2_ fluctuations without the influence of other factors that covary in situ (i.e., temperature, wave exposure). We then combined gene expression and physiological analyses with scanning electron microscopy (SEM) to examine the impact of pCO_2_ variability on coral biomineralisation. Our comparative approach linking processes across biological scales uncovers differences in gene expression regulation between corals from the environmentally distinct habitats, revealing an enhanced capacity to cope with ocean acidification gained via exposure to environmental variability.

## 2 Materials and Methods

### 2.1 Study Location

The experiment was performed over 8 weeks in the austral summer (January–March 2021) at Heron Island Research Station (HIRS), southern Great Barrier Reef. This study focused on two environmentally distinct habitats, the reef flat and reef slope (Figure 1). Tidally‐driven fluctuations in seawater temperature and pCO_2_ result in greater environmental variability on the reef flat, fluctuating up to 4.3°C day^−1^ and 797 μatm day^−1^ versus 1.3°C day^−1^ and 399 μatm day^−1^ on the reef slope (Figure 1; Brown et al. 2022). In‐field measurements (temperature, photosynthetically active radiation (PAR), and nutrients) were recorded concurrently with the mesocosm experiment at the same locations where corals were collected (8 January—18 March 2021). Seawater pCO_2_ was recorded over the same season, but in 2016 (8 January—18 March 2016; Brown et al. 2022). The mean seawater pCO_2_ (μatm) in situ was similar between the reef flat (454 ± 3.0) and reef slope (418 ± 1.9), but the reef flat experienced twice the mean daily pCO_2_ amplitude than the reef slope (Figure 1). A number of other environmental conditions covaried with pCO_2_ between the two habitats, including temperature and PAR, but not seawater nutrients (ammonium, nitrate, nitrite, phosphate; Brown et al. 2022). Additional details on environmental conditions within the two habitats are described in Brown et al. (2022).

### 2.2 Experimental Design and Physiological Analyses

Fragments of the coral *Pocillopora damicornis* were collected from the reef flat and slope locations within the same depth range (1–3 m) in January 2021 (Figure 1; Brown et al. 2022). Four fragments were collected from each individual colony (genetic clones), totaling 96 fragments from 24 colonies (*n* = 12 colonies per habitat). All coral colonies used in the experiments were confirmed to be *Pocillopora damicornis* (GenBank Accession numbers OP296503–OP296521; 100% match to *Pocillopora* type alpha cf. (Schmidt‐Roach et al. 2013) with GenBank accession numbers JX985598 and JX985606; (Brown et al. 2022)). ITS2 rDNA data, coupled with the phylogenetic analyses of psbA sequences (Genbank Accession numbers OP279755–OP279774), confirmed that all coral specimens contained *Cladocopium latusorum* (Turnham et al. 2021; Brown et al. 2022), recently described as a pocilloporid‐specific endosymbiont (Turnham et al. 2021).

Corals were exposed to two distinct treatments for 8 weeks: (1) stable seawater pCO_2_ or (2) variable seawater pCO_2_ (Figure 1). Seawater pCO_2_ was continuously recorded in experimental treatment sumps, with a 4.2‐fold difference in mean diel pCO_2_ amplitude (μatm) between the variable (911.3 ± 30.69) and stable (218.3 ± 9.14) treatments across the experimental period (Figure 1). Temperature, irradiance, and nutrients did not differ within or across experimental treatments (Brown et al. 2022). The mean temperature was 27.4°C and the mean PAR was ~125 μmol quanta m^2^ s^−1^ throughout the experiment. Additional details on experimental design and conditions are described in (Brown et al. 2022).

Net calcification was determined by comparing the buoyant weight at the start and end of the 8‐week experiment using the method described by (Davies 1989). At the end of the experiment, metabolic rates (net photosynthesis, dark respiration and light‐enhanced dark respiration) were assessed via changes in oxygen evolution (Brown et al. 2022). At the same time, several chips per colony were preserved in 1 mL of RNA*later* stabilisation solution (Thermo Fisher AM7021). In addition, half of the coral fragments (2 per colony per treatment; *n* = 48 total) were flash frozen in liquid nitrogen and used to quantify the following physiological properties: calcium carbonate (CaCO_3_) density, host tissue soluble protein, mycosporine‐like amino acids, endosymbiont density, and chlorophyll *a* concentration following the methodology in (Brown et al. 2022). The other half of the fragments were transported alive to the Australian Cancer Research Foundation's Cancer Biology Imaging Facility at the University of Queensland to assess intracellular acid–base status and acidification resilience (i.e., acidosis magnitude and rate of pHi recovery) following established methods (Innis et al. 2021). Additional details on all physiological analyses are described in (Brown et al. 2022).

### 2.3 Skeletal Micromorphological Analysis

**FIGURE 2:**
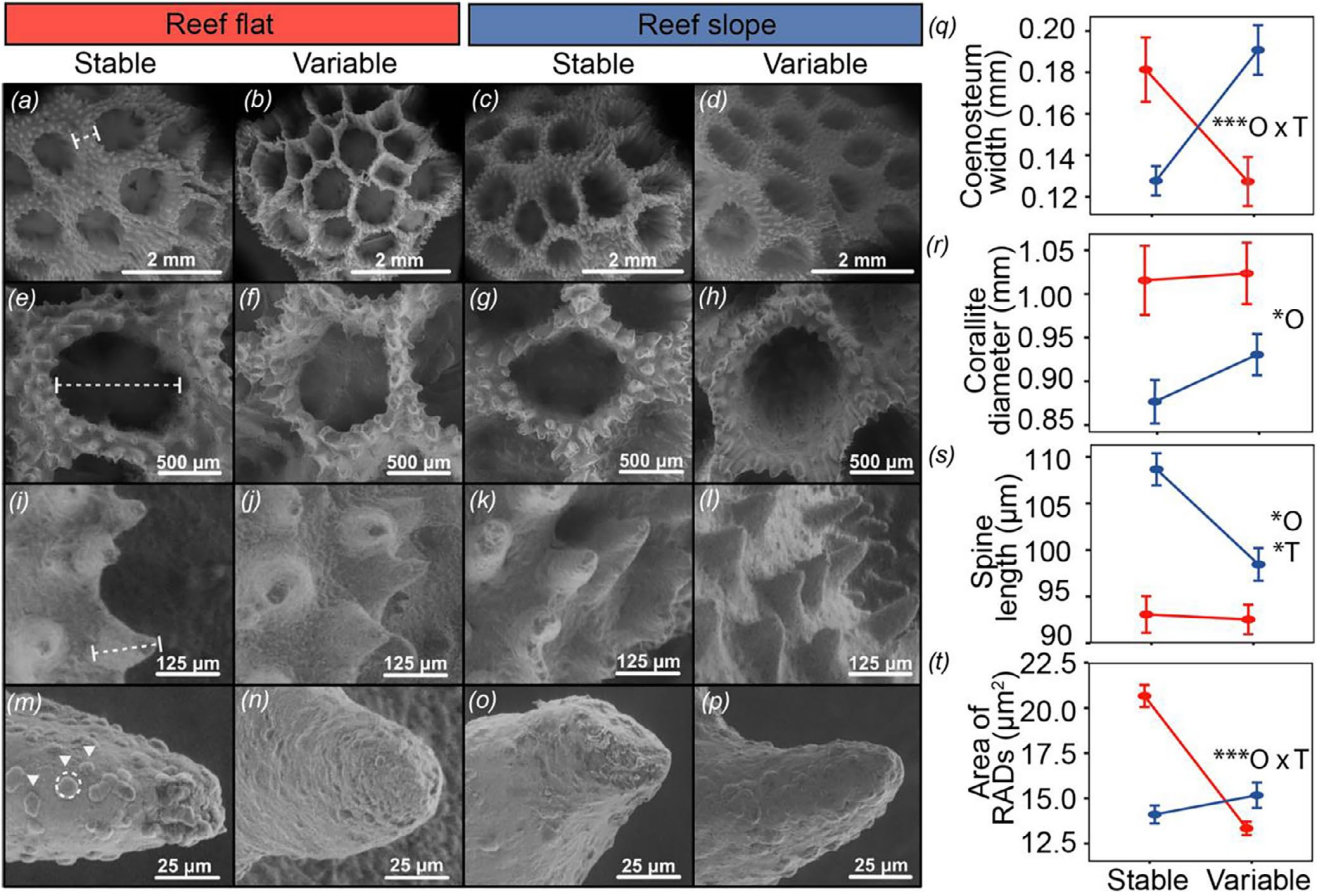
Comparison of skeletal morphometrics of *P. damicornis* across origin and treatment conditions. Surface morphology imaged by scanning electron microscopy (SEM) for features of interest: (a–d) an overall view of the skeleton (56×), (e–h) individual whole calyxes (124×), (i–l) length of spine structures between the calyxes (141×), and (m–p) surface area of rapid accretion deposits (RADs; globular elements indicated by white triangles in panel m) (1013×). (q) Distance between (b/t) corallites by origin and treatment, where dashed lines in (a) indicate an example measurement. (r) Corallite diameter by origin and treatment, where dashed lines in (e) indicate an example measurement. (s) Spine length by origin and treatment, where dashed lines in (i) indicate an example measurement. (t) Area of RADs by origin and treatment, where triangles and dashed circle in (m) indicate individual RADs and an example measurement, respectively. All data are displayed as means ±SE. Insets indicate statistical significance (*p < 0.05, **p < 0.001, ***p < 0.0001) of individual and interactive effects for origin (O) and treatment (T) as determined from linear mixed effects models. Reef flat origin, red; reef slope origin, blue. Treatment is indicated by column (a–p) or on the x‐axis (q–t). Dashed lines indicate measurements.

The limited amount of new CaCO_3_ deposition observed during the 8‐week exposure (~15%–30% for each fragment; Brown et al. 2022) precluded our resolution to detect changes in net calcification or CaCO_3_ density of newly formed skeleton that were attributable to experimental pCO_2_ treatment conditions. To better resolve changes in biomineralisation resulting from the seawater pCO_2_ variability treatments, a total of 16 coral fragments (*n* = 4 per origin per treatment) were selected for skeletal micromorphological analyses. All tissue was removed from the skeletons by soaking the fragments in 10% sodium hypochlorite for 24 h, rinsing with DI water, and drying. Areas of CaCO_3_ deposition that occurred during the experiment were identified by comparing images at the start and end of the 8‐week experiment (Figure S1). These deposits of new CaCO_3_ were carefully chipped off of the experimental fragments using a razor blade and imaged using a scanning electron microscope (SEM; Quanta 600 FEG Mark II Environmental Scanning Electron Microscope, Field Electron, and Ion Company). Using the SEM, fragments were imaged across scales with magnification maintained between samples: an overall view of the skeleton (56×), individual whole calyxes (124×), spine structures between (141×) and inside (164×) the calyxes, and the rapid accretion deposits (RADs) on the spines (1013×; Figure 2). Several features of interest previously used to investigate coral biomineralisation (Scucchia et al. 2023; Scucchia, Malik, Zaslansky, et al. 2023) were quantified using ImageJ (v1.53c) (Schneider, Rasband, and Eliceiri 2012), including: number of corallites, distance between corallites (i.e., coenosteum width), corallite diameter, circularity of the corallite, number of spines within calyx, spine length and maximum spine width (on spines both between and inside the calyx), number of RADs, and size of RADs. The significant interaction between treatment and origin was explored on all micromorphological features using linear mixed effects models, with colony as a random effect. The significance of fixed effects and their interactions was determined using an analysis of variance with a type III error structure using the Anova function in car package (Fox et al. 2012). Significant interactive effects were followed by pairwise comparison of estimated marginal means using the emmeans package with Tukey HSD adjusted *p* values (Lenth et al. 2018). Data were tested for homogeneity of variance and normality of distribution through graphical analyses of residual plots for all models. All statistical analyses were done using R version 4.0.3 software (R Core Team 2021), and graphical representations were produced using the package ggplot2 (Wickham 2016).

### 2.4 RNA and DNA Extractions

Samples for nucleic acid extraction were preserved in RNA*later* stabilisation solution and stored at −80°C until extraction. Genomic DNA and total RNA were extracted concurrently using the Zymo Quick‐DNA/RNA Miniprep Plus Kit (Zymo Research #D7003) using the following modifications to the manufacturer's protocol. Samples were thawed on ice, and two small fragments (5 mm × 5 mm) were removed from the stabilisation solution using sterile forceps. Immediately, excess stabilisation solution was removed with the corner of a KimWipe, and fragments were submerged in a new 1.5 mL screw‐cap tube containing 0.5 mm glass beads (Fisher Scientific, #50‐212‐143) and 800 μL of DNA/RNA shield (Zymo Research, #R1100‐50). Samples were homogenised by vortex at max speed for 1–2 min. Following homogenisation, 400 μL of homogenate was removed and centrifuged for 3 min at 9000 rcf. The supernatant was transferred to a new tube and mixed with 30 μL Proteinase K digestion buffer and 15 μL Proteinase K (Zymo Research #D7003). This mixture was incubated at room temperature for 15 min, followed by another centrifugation step for 3 min at 9000 rcf. The supernatant was combined with an equal volume of DNA/RNA lysis buffer (Zymo Research #D7003). Extraction was thereafter performed as described by the manufacturer's protocol, including the optional DNase I treatment. DNA and RNA concentrations were quantified using the Qubit dsDNA and RNA Broad Range kits (Invitrogen #Q10211), and integrity was assayed using non‐denaturing gel electrophoresis (1.5% agarose gel, 60 min at 60 V). Purity was assessed using Nanodrop.

### 2.5 Species Identification

Twenty of the 24 colonies were previously identified as *P. damicornis* (Brown et al. 2022). For the four colonies that were not identified due to difficulties with PCR amplification, the mitochondrial open reading frame (mtORF) region was amplified from new genomic DNA extracts via PCR as described by Burgess et al. (2021); Johnston, Forsman, and Toonen (2018) using primers from Flot et al. (2008): FatP6.1 (5′‐TTTGGGSATTCGTTTAGCAG‐3′) and RORF (5′‐SCCAATATGTTAAACASCATGTCA‐3′). PCR master mixes contained 12.55 μL of EmeraldAmp GT PCR Master Mix (TaKaRa Bio USA Inc. Cat # RR310B), 0.32 μL of forward and reverse primers as listed, 1 μL of template DNA, and 10.80 μL of nuclease‐free water, totaling 25 μL of final volume. Negative controls were included as master mix without template DNA. mtORF was amplified using a polymerase chain reaction (PCR) profile of a single denaturation set of 94°C for 60 s followed by 30 cycles of 94°C for 30 s for denaturation, 53°C for 30 s for annealing, and 72°C for 75 s for extension and a final incubation of 72°C for 5 min. PCR products were assessed with a 1.5% agarose gel in TAE for 30 min at 80 V with an expected band size of approx. 1000 bp. PCR products were cleaned using ethanol precipitation. 1/10th of the volume of 3 M sodium acetate (Fisher Cat. AAJ61928AE) was added to each PCR product, followed by an addition of 3 times the total volume of the mixture of ice‐cold 100% ethanol. The mixture was incubated overnight at −20°C and DNA was precipitated by centrifugation at 15,000 rcf for 30 min at room temperature. The pellet was washed with 70% ethanol twice, dried, and resuspended in 30 μL of 1 M Tris‐HCI, pH 8.0 (Fisher Cat. 15568025). DNA quantity was assessed using Broad Range dsDNA Qubit and Nanodrop. Sanger sequencing using the same primers utilised during PCR amplification was performed at the URI Genomics and Sequencing Center using Applied Biosystems BigDye Terminator v3.1. Geneious Prime (Version 2023.2.1) was used to align the four sequences (Muscle 5.1 alignment, PPP algorithm, default parameters) to known *P. damicornis* (NCBI Accession Numbers: JX994077, KJ720219, JX994086, JX624991, KF583925, KF583946, EU374235, KJ720218, KP698587, KM215098, KX538982, KJ720226, KX538983, KF583950, JX994087, KJ720235, JX985618, KJ690905, JX625025, and KX538984) and *P. acuta* (NCBI Accession Numbers: JX994073, JX624999, KF583928, KF583935, EU374226, FJ424111, KJ720240, KM215075, KJ690906, KP698585, KX538985, KX538986, KM215104, and KJ720241) mtORF sequences and a Neighbour‐Joining tree was built from the alignment. The samples sequenced in this study were identified as *P. damicornis* .

### 2.6 Library Preparation and Tag Seq Analysis

Extracted total RNA was prepared for TagSeq (Lohman, Weber, and Bolnick 2016). Library preparation and sequencing of the 48 samples were conducted at the University of Texas at Austin, Genomic Sequencing and Analysis Facility. Sequencing was completed targeting standard coverage of 3–5 million 100‐bp single‐end reads per sample (Illumina NovaSeq 600 SR100). We used fastp to trim raw TagSeq reads of TruSeq 1 Illumina adapters, poly‐A and poly‐G sequences, and remove low‐quality reads (i.e., reads with > 40% of bases with Phred score < 30) and low‐complexity reads (i.e., < 50%; Chen et al. 2018). Before and after filtering, MultiQC was used to assess filtering success (Ewels et al. 2016). After successful trimming and filtering, filtered reads were mapped to the *Pocillopora damicornis* genome (Cunning et al. 2018) and *P. acuta* genome (Stephens et al. 2022) using HISAT2 using the downstream‐transcriptome‐assembly mode for unpaired reads (Kim et al. 2019). Mapping rate was approximately 150% higher for the *P. acuta* genome than the *P. damicornis* genome (average of 72.48% compared to 29.10%), despite all corals used in the experiments confirmed as *P. damicornis* (Brown et al. 2022). Accordingly, downstream analyses were performed using the *P. acuta* genome. Mapped reads were quantified and assembled using StringTie (Pertea et al. 2016) and a gene count matrix was generated using the Stringtie script prepDE.py for downstream analysis. All code and data are publicly available (https://github.com/imkristenbrown/Heron‐Pdam‐gene‐expression/). All raw TagSeq data can be accessed at the Sequence Read Archive (SRA) (https://www.ncbi.nlm.nih.gov/sra/PRJNA934298).

### 2.7 Quality Control of Gene Expression Data

All analyses were conducted using R version 4.0.3 software (R Core Team 2021). First, all genes not detected (i.e., 100% of the samples showed counts of 0) across any of our samples (*n* = 48) were removed, leaving data for 24,220 genes of the 33,730 genes in the *P. acuta* genome. Next, low‐expression genes, defined as any gene that did not have at least 10 counts in 25% of the samples, were removed using the function pOverA in the package *genefilter* (Gentleman et al. n.d.). This retained genes that were expressed in at least one of the four different conditions, leaving a robust dataset of counts for 9,056 genes. Gene counts were normalised and transformed using variance stabilising transformation (herein referred to as ‘vst‐normalized gene expression’) in the package *DESeq2* (Love, Huber, and Anders 2014). After examination of vst‐normalised gene expression plots, two outliers were identified (two samples of the same genotype, RF16). These outliers were also identified as having high percentage of sequence duplication based on FastQC of raw fastq sequence files and were subsequently removed from the analysis (Figure S2). Following the removal of these two samples, pOverA filtering and variance stabilising transformation was re‐run, and the final dataset for statistical analysis included a robust dataset of counts for 9,012 genes from 46 samples. The resulting sample group sizes (biological replicates) for gene expression analysis were: flat‐stable (*n* = 11), flat‐variable (*n* = 11), slope‐stable (*n* = 12) and slope‐variable (*n* = 12).

### 2.8 Co‐Expression Analysis

Weighted Gene Co‐expression Network Analysis (WGCNA, (Langfelder and Horvath 2008)) was carried out using the R package WGCNA (version 1.72–5) to identify co‐expression patterns based on native habitat of origin (flat vs. slope) or 8 weeks of exposure to pCO_2_ treatments (stable vs. variable). A signed adjacency similarity matrix of vst‐normalised gene expression was constructed for all gene pairs across samples using the adjacency function (using a soft power of 5), then converted into a topological overlap dissimilarity matrix using the function TOMsimilarity (Langfelder and Horvath 2008). Next, a hierarchical clustering of genes based on topological overlap was performed using the function hclust, followed by module identification using the function cutreeDynamic (hybrid method, deepSplit = 1) in the package *dynamicTreeCut* (Langfelder, Zhang, and Horvath 2008), retaining modules with at least 30 genes and merging highly similar modules using the function mergeCloseModules with a cut height of 0.15 (eigengenes correlated at *R* > 0.85). Trait data (categorical and physiological metrics) were then related to the expression of modules and clustered based on eigengene correlation using the package *complexHeatmap* (Gu, Eils, and Schlesner 2016). The complete list of the caterogrical and physiological metrics included was: net calcification, CaCO_3_ density, net photosynthesis, light‐enhanced dark respiration, dark respiration, photosynthesis:respiration ratio, protein concentration, symbiont density, chlorophyll *a* concentration, mycosporine‐like amino acid shinorine, rate of pHi recovery (symbiocyte and non‐symbiocyte), acidosis magnitude (symbiocyte and non‐symbiocyte), as well as categorical factors treatment, origin and genotype.

Gene ontology (GO) enrichment was explored for WGCNA modules using the EggNog and KEGG functional annotation files from the *P. acuta* genome (Stephens et al. 2022). Of the 9,012 genes in the dataset, there was no GO annotation for 4,448 of the genes and no KEGG annotation for 3,828 of the genes. Gene length information was calculated from the “gff3” file of the *P. acuta* genome (Stephens et al. 2022). An “over‐represented *p*‐value” for each GO term was calculated using the Wallenius method in the package *goseq* (Young et al. 2012) and only the biological process (BP) ontology was investigated. GO terms were considered to be overrepresented if they had a *p*‐value of less than 0.01. An adjusted *p*‐value using the Benjamini & Hochberg (“BH”) method of the *p*.adjust function in R was calculated for each GO term and is available in the Data S1. The overrepresented GO term set was further reduced using a similarity matrix with a threshold of 0.7 to collapse similar GO terms for plotting them by their parent terms using the package *rrvgo* (Sayols 2023).

### 2.9 Differentially Expressed Genes and Gene Expression Frontloading

A linear mixed effects model was used to analyse differential gene expression by origin and treatment, with colony as a random effect, using the package *glmmSeq* (Lewis et al. 2021). First, a dispersion vector and size factors were calculated for each gene using *DESeq2* on the raw gene count matrix (i.e., vst‐normalised gene counts were not used as input for *glmmSeq* analysis). Then, the interactive effects of origin and treatment were explored on gene expression data (raw count data with dispersions provided), with colony genotype as a random effect. For one gene, the model did not converge, causing the final dataset for *glmmSeq* to contain 9,011 genes. Adjusted *p*‐values were calculated using the package *qvalue* (Storey et al. 2023). Genes with an adjusted *p*‐value (*q*‐value) < 0.05 were considered to be differentially expressed.

Because habitat of origin was a major driver of the gene expression responses, we further tested for frontloading of transcripts (constitutive levels of expression) following the methodology of (Gurr et al. 2022). We calculated two ratios: a control ratio (stable flat/stable slope) and a fold change ratio (variable/stable flat/variable/stable slope; Gurr et al. 2022). “Frontloaded” transcripts are defined as having: (i) a greater expression in the flat habitat of origin in the variable treatment compared to the slope origin corals in the stable treatment (control ratio > 1) and (ii) a smaller fold change from variable to stable in the flat origin compared to the slope origin (fold change ratio < 1; Barshis et al. 2013). A fold change ratio of < 1 indicates that these transcripts underwent less of an expression change from the stable to variable treatment in the corals from the flat habitat, suggesting “frontloading” of expression to cope with a more variable environment (Barshis et al. 2013), since these genes were constitutively higher expressed in the flat origin corals under a stable pH treatment (control ratio > 1). These calculations were made based on the *glmmseq* estimated mean expression of each gene in each treatment:origin combination based on the fitted model.

GO enrichment was performed for genes differentially expressed by origin, treatment, and the interaction as well as for frontloaded genes using the same method as described for the WGCNA modules. Enrichment of these gene sets was tested against the 9,011 genes in the glmmSeq dataset. Full datafiles are available in the Data S1.

### 2.10 Expression of Putative Biomineralisation‐Related Genes

The expression of coral biomineralisation‐related genes from the literature (Scucchia, Malik, Putnam, et al. 2021; Scucchia, Malik, Zaslansky, et al. 2021) was compared to both the differential expression and frontloaded results. A BLASTp search (using BLAST+ 2.9.0‐iimpi‐2019b) was performed against a database of the *P. acuta* genome to identify the best match (*e*‐value < 0.01) of each of these coral biomineralisation‐related genes in the genome of *P. acuta* . The top hit for each biomineralisation‐related gene was used (Stephens et al. 2022).

## 3 Results

### 3.1 Skeletal Micromorphological Analysis

The distance between corallites, corresponding to forming the coenosteum, was significantly influenced by the interaction between treatment and origin (*Χ* ^2^ = 27.4, *p* < 0.0001; Figure 2a–d,q). Post hoc analyses revealed that for *P. damicornis* originating from the variable reef flat, coenosteum width was thinnest under native variable pCO_2_ conditions (0.12 mm; Figure 2a,b,q), increasing in thickness by ~40% when exposed to stable pCO_2_ conditions (0.18 mm; Figure 2a; *p* = 0.005). Corals that originated from the reef slope showed the opposite pattern, increasing coenosteum thickness relative to native stable conditions in response to variable pCO_2_ conditions (*p* = 0.0006; Figure 2c,d,q). Corallite diameter was significantly influenced by the individual effect of origin (*Χ* ^2^ = 5.3, *p* = 0.02), but not treatment (*Χ* ^2^ = 0.05, *p* = 0.81; Figure 2e–h,r). Corallite diameter was greatest in corals originating from the reef flat and did not change in response to treatment (1.02 mm, Figure 2e,f,r), whereas in corals from the reef slope, corallite diameter trended wider under non‐native variable pCO_2_ conditions compared to native stable conditions (0.93 vs. 0.88 mm, respectively; Figure 2g,h,r). The length of the spines on the coenosteum between the calyces was significantly influenced by origin (*Χ* ^2^ = 4.9, *p* = 0.03) and treatment (*Χ* ^2^ = 4.8, *p* = 0.03; Figure 2i–l,s). The longest spines were observed in *P. damicornis* originating from the reef slope that were exposed to their native stable pCO_2_ conditions (109 μm; Figure 2k,l,s), which became 10% shorter in response to variable pCO_2_ conditions (98 μm; Figure 2k,l,s), suggesting the reduction of skeletal development. Yet, corals that originated from the reef flat had the shortest spines, which did not change in response to treatment (93 μm; Figure 2i,j,s). In contrast, within the calyx spine length (*Χ* ^2^ = 0.8, *p* > 0.27) and maximum spine width (*Χ* ^2^ = 0.8, *p* > 0.27) showed no significant direct or interactive differences by origin or treatment. No significant differences were found in the number of corallites area^−1^ (*Χ* ^2^ = 0.1, *p* > 0.70), circularity of the corallite (*Χ* ^2^ = 1.7, *p* > 0.20), or the number of spines within the calyx (*Χ* ^2^ = 2.2, *p* > 0.14; Figure S3).

The terminal portion of the skeletal spines within the coenosteum were imaged at higher magnification to focus on areas of the skeleton with rapid CaCO_3_ deposition (Drake et al. 2020)—the RADs (globular elements in Figure 2m–p). The size (area) of the RADs was significantly influenced by the interaction between treatment and origin (*Χ* ^2^ = 27.4, *p* < 0.0001; Figure 2t). Post hoc analyses revealed that *P. damicornis* originating from the reef flat had smaller RADs when exposed to their native variable pCO_2_ conditions (13 μm; Figure 2n,t), increasing by ~60% in response to non‐native stable pCO_2_ (21 μm; Figure 2m,t; *p* < 0.0001), suggesting the promotion of skeletal development. In contrast, the area of RADs for corals from the reef slope was not affected by treatment (Figure 2o,p,t). The number of RADs per spine was significantly influenced by the individual effects of origin (*Χ* ^2^ = 9.8, *p* = 0.002) and treatment (*Χ* ^2^ = 4.4, *p* = 0.04). Reef slope corals had significantly fewer RADs compared to corals from the reef flat (Figure 2m–p). For corals originating from the reef flat, the number of RADs per spine was lowest under native variable pCO_2_ conditions (21 RADs per spine), and increased by ~40% to 30 RADs per spine in non‐native stable pCO_2_ conditions (Figure 2m,n).

### 3.2 Functional Enrichment of Differentially Expressed Genes

Of the 9,012 genes in the dataset, 840 were differentially expressed by origin, 18 were differentially expressed by treatment, and 30 were differentially expressed by the interaction of treatment and origin. Only two of the 30 differentially expressed genes by the interactions of origin and treatment were not also significant by the individual effects of origin or treatment alone. One of these genes, a homologue of “Caspase, interleukin‐1 beta converting enzyme (ICE)”, had significantly higher expression in corals exposed to their native pCO_2_ conditions; that is, under stable pCO_2_ conditions for corals originating from the reef slope and under variable pCO_2_ conditions in corals from the reef flat. An additional three genes were differentially expressed by treatment and the interaction between origin and treatment, but not by origin. Again, two of these genes, “GDP‐fucose synthetase, extended (e) SDRs” and “GT4_PimA‐like (phosphatidyl‐myo‐inositol mannosyltransferase)”, had higher expression in corals exposed to their native pCO_2_ conditions. Only one gene had consistently higher expression in the variable pCO_2_ treatment regardless of origin, and was annotated as belonging to the Isy1‐like splicing family.

GO enrichment of differentially expressed genes showing a significant interaction of treatment and origin was performed by comparing the GO terms for the 30 differentially expressed genes (739 terms) to all GO terms in the 9011‐gene dataset (17,742 terms). A total of 81 BP ontology GO terms were overrepresented (*p* < 0.05). The highest rank overrepresented terms included “regulation of dopamine uptake involved in synaptic transmission” (GO:0051584; GO:0051586), “regulation of catecholamine uptake involved in synaptic transmission” (GO:0051940; GO:0051944), “dolichol biosynthetic process” (GO:0019408), “coenzyme A catabolic process” (GO:0015938), and “nucleoside bisphosphate catabolic process” (GO:0033869). A total of 14 KEGG terms were identified as overrepresented in the 30 differentially expressed genes including: “potassium voltage‐gated channel Eag‐related subfamily H member 8” (K04911), “translation initiation factor eIF‐2B subunit gamma” (K03241), “palmitoyltransferase ZDHHC1/11” (K20027), “HMG box transcription factor 1” (K21644), and “pre‐mRNA‐splicing factor ISY1” (K12870). GO enrichment by the individual effects of origin and treatment are described in the Data S1 (Results: Data S1; Figure S4).

### 3.3 Gene Co‐Expression Modules

**FIGURE 3:**
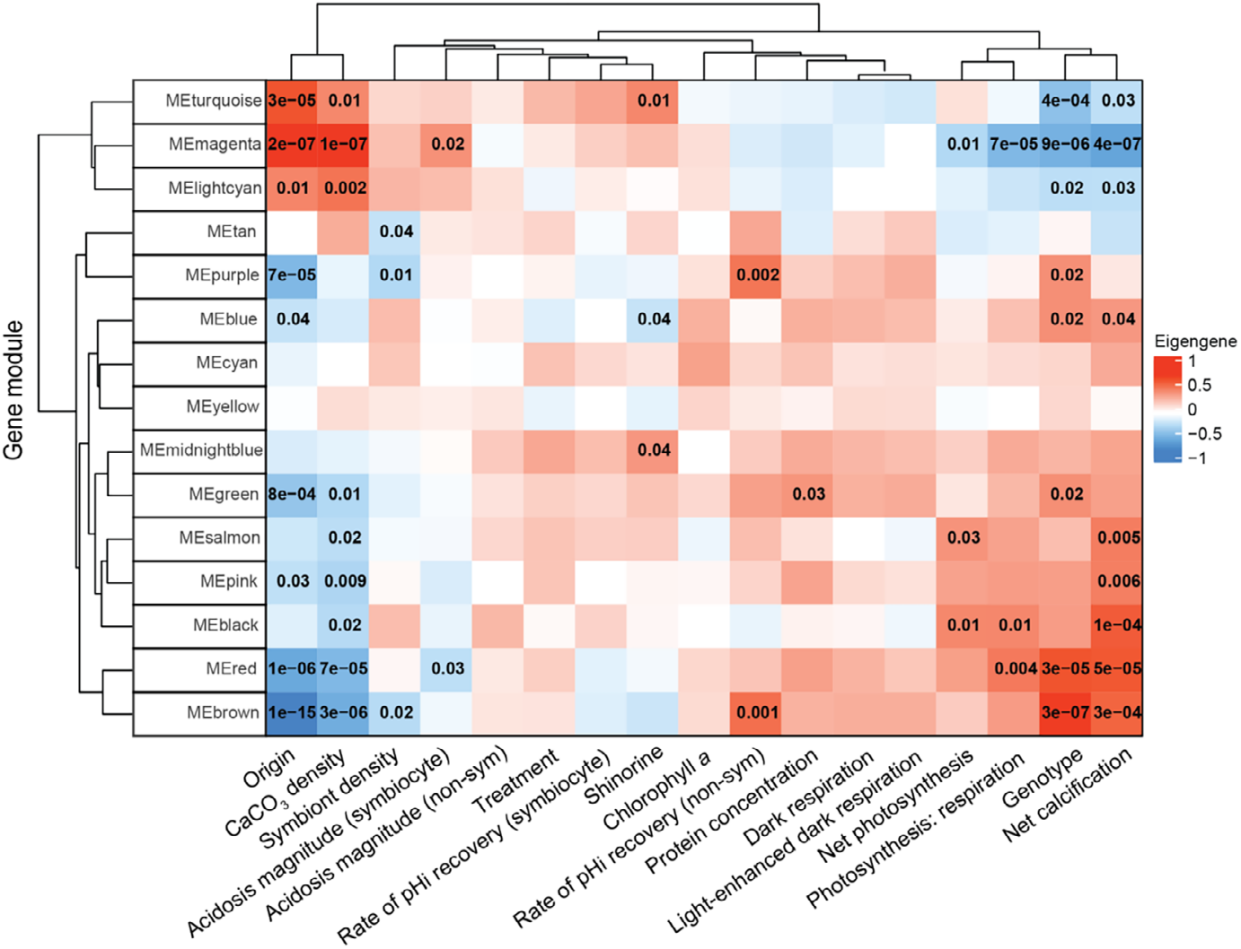
Weighted gene co‐expression network analysis (WGCNA) module‐trait relationships. WGCNA analysis with modules of genes (named as MEcolor) correlated with traits, where the colour is determined by the strength and direction (positive eigengene expression: Red, negative eigengene expression: Blue) of the correlation between the gene module and trait. Modules and traits are ordered by hierarchical clustering. Bold values indicate a module is significantly related to the respective trait (*p* ≤ 0.05). ‘Origin’ and ‘Treatment’ are binary categorical traits, indicating site of origin (reef flat =  1, reef slope = 0) and pCO_2_ treatment (variable = 1, stable = 0). CaCO_3_, calcium carbonate; non‐sym, non‐symbiocyte; pHi, intracellular pH.

Network analysis resulted in 15 co‐expression modules of 65 to 2,558 genes each, named with a colour based on the convention of (Langfelder and Horvath 2008). The expression pattern of three modules demonstrated significant positive correlation with origin: MEturquoise (*n* = 2,558 genes, *p* < 0.0001), MEmagenta (*n* = 219, *p* < 0.0001), and MElightcyan (*n* = 65, *p* = 0.01). These three modules also demonstrated significant positive correlation with CaCO_3_ density, which was significantly higher in *P. damicornis* originating from the reef slope (Figure 3; Brown et al. 2022). Two modules, MEred (*n* = 425 genes, *p* < 0.0001) and MEbrown (*n* = 942 genes, *p* < 0.0001), demonstrated the opposite pattern by origin and CaCO_3_ density, reflecting expression patterns that were significantly correlated with traits associated with *P. damicornis* that originated from the reef flat (Figure 3). Interestingly, these reef flat‐associated modules showed eigengene expression patterns that were significantly positively correlated with net calcification (*p* < 0.0001). In fact, a total of six modules (totalling 4,126 genes) demonstrated expression patterns positively correlated with net calcification, including MEred (*p* < 0.0001) and MEbrown (*p* = 0.0003), but also MEblack (*n* = 396 genes, *p* < 0.0001), MEpink (*n* = 220, *p* = 0.006), MEsalmon (*n* = 154, *p* = 0.005), and MEblue (*n* = 1,989, *p* = 0.04). By hierarchical clustering of trait values (columns, Figure 3), net calcification most closely clustered to genotype and the metabolic traits of photosynthesis:respiration ratio (P:R) and net photosynthesis, with all traits showing the same trend of positive correlation with expression of those modules (MEbrown, MEred, MEblack, MEpink, MEsalmon, and MEblue). None of the modules showed significant correlation between eigengene expression and treatment (Figure 3).

**FIGURE 4:**
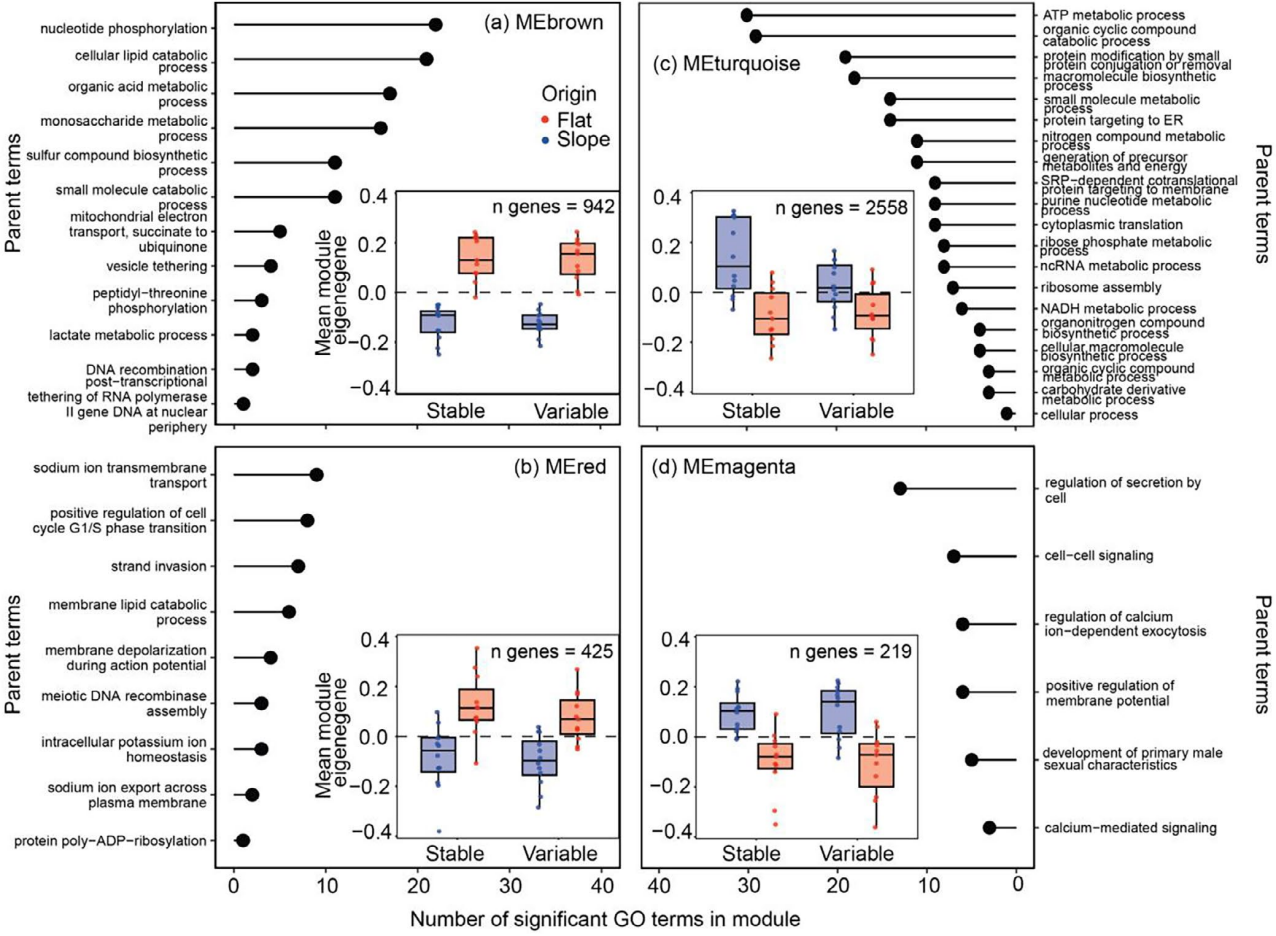
Gene ontology enrichment analysis of WGCNA modules significantly related to calcification. Number of overrepresented gene ontology (GO) terms are plotted by parent GO categories from the modules that displayed positive expression in calcification, (a) MEbrown (*p* < 0.01) and (b) MEred (*p* < 0.01), or negative expression in calcification, (c) MEturquoise (*p* < 0.001) and (d) MEmagenta (*p* < 0.01). Inset displays the mean module eigengene expression by origin and treatment and the number of genes (n genes) in each module.

GO enrichment analysis was conducted for two WGCNA modules (MEbrown and MEred) with an expression pattern that showed highly significant positive correlation with net calcification and significant negative correlation with origin (i.e., the genes in these modules were higher expressed in the reef flat; Figure S5). A total of 206 GO terms in the BP ontology were found to be overrepresented (*p* < 0.05) in the MEbrown module, which grouped into 30 parent terms (Figure 4a). The highest rank terms in this module included: “peptidyl‐threonine phosphorylation” (GO:0018107), “organic acid metabolic process” (GO:0006082), and “oxoacid metabolic process” (GO:0043436). The most abundant parent terms were: 1. nucleotide phosphorylation, 2. cellular lipid catabolic process, and 3. organic acid metabolic process (Figure 4a). A total of 209 GO terms in the BP ontology were found to be overrepresented (*p* < 0.05) in the MEred module, which grouped into 26 parent terms (Figure 4b). The highest rank terms in this module included: “sodium ion transmembrane transport” (GO:0035725), “regulation of resting membrane potential” (GO:0060075), and “regulation of glucocorticoid metabolic process” (GO:0031943). The most abundant parent terms were: (1) sodium ion transmembrane transport, (2) monoatomic cation transmembrane transport, (3) positive regulation of cell cycle G1/S phase transition, and (4) S‐adenosylhomocysteine metabolic process (Figure 4b).

GO enrichment was also conducted for two WGCNA modules (MEturquoise and MEmagenta) with an expression pattern that showed highly significant negative correlation with net calcification and positive correlation with origin (i.e., the genes in these modules were higher expressed in the reef slope, regardless of treatment; Figure S6). A total of 382 GO terms in the BP ontology were found to be overrepresented (*p* < 0.05) in the MEturquoise module, which grouped into 46 parent terms (Figure 4c). The highest rank terms in this module included: “cytoplasmic translation” (GO:0002181), “translation” (GO:0006412), and “peptide biosynthetic process” (GO:0043043). The most abundant parent terms were: (1) ATP metabolic process, (2) organic cyclic compound catabolic process, and (3) protein modification by small protein conjugation or removal (Figure 4c). A total of 210 GO terms in the BP ontology were found to be overrepresented (*p* < 0.05) in the MEmagenta module, which grouped into 23 parent terms (Figure 4d). The highest rank terms in this module included: “calcium‐mediated signalling” (GO:0019722), “cell–cell signalling” (GO:0007267), and “T‐tubule organisation” (GO:0033292). The most abundant parent terms were: 1. regulation of secretion by cell, 2. cell–cell signalling, and 3. regulation of calcium ion‐dependent exocytosis (Figure 4d).

### 3.4 Frontloading of Gene Expression

**FIGURE 5:**
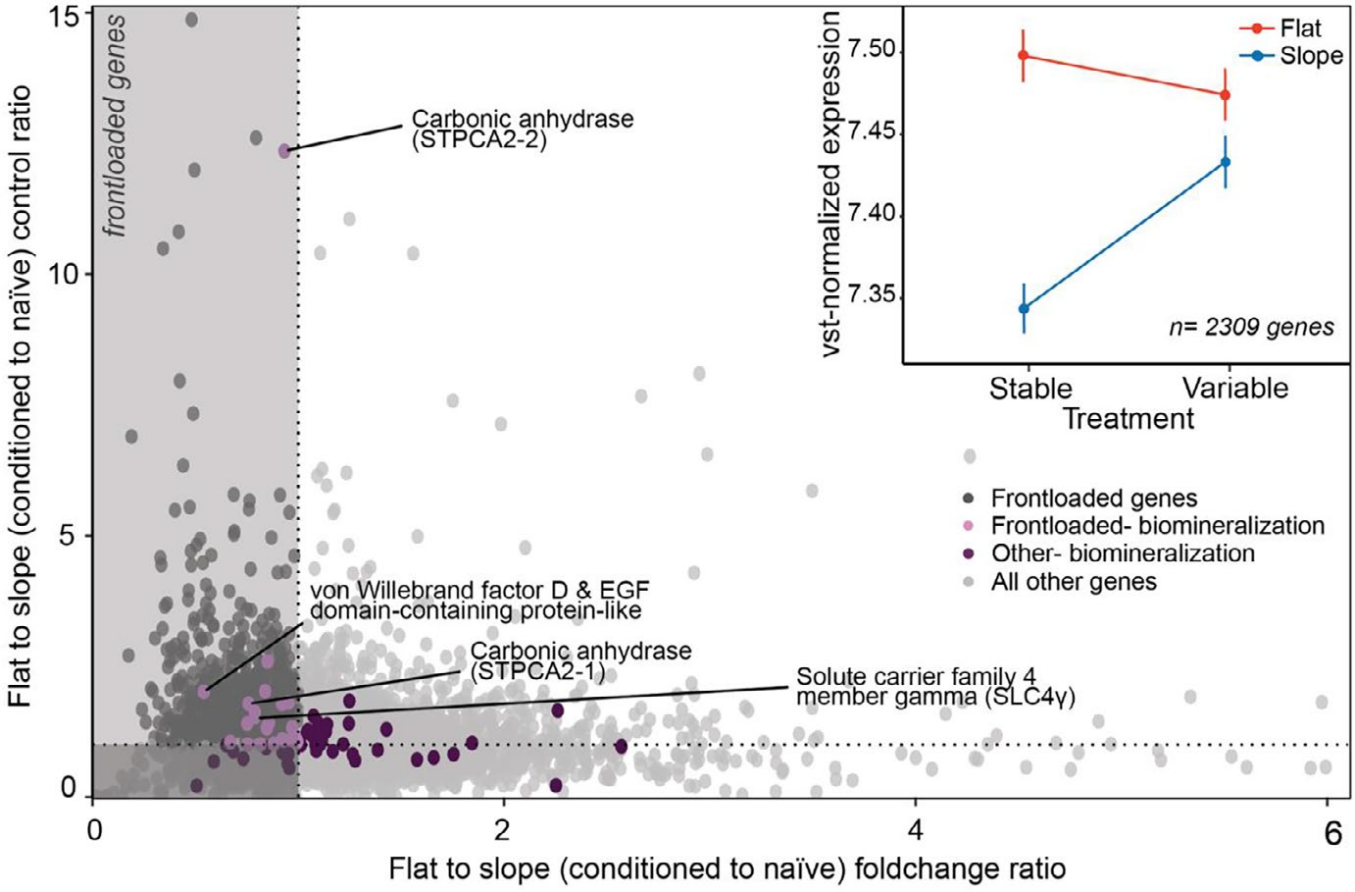
Constitutive upregulation of gene expression indicates frontloading of key biomineralisation‐related genes. Frontloaded genes, as dark grey points in the upper left quadrant, with putative biomineralisation‐related genes displayed in purple. Inset displays the vst‐normalised gene expression (mean ± SE) of frontloaded genes by origin and pCO_2_ treatment.

A total of 2,309 out of 9,012 genes showed a control ratio > 1 and a fold change ratio < 1 (Figure 5), which meets the criteria for “frontloaded” transcripts (Barshis et al. 2013). By definition, these transcripts have consistently higher expression in corals originating from the reef flat and show an upregulation of expression in corals from the reef slope when exposed to the variable treatment (Figure 5). Using the same GO enrichment approach as for the differentially expressed gene set, GO terms from the 2,309 frontloaded genes (11,760 terms) were compared to all GO terms in the 9,012 gene dataset (17,742 terms). A total of 471 terms of BP ontology were overrepresented. The highest rank overrepresented terms included: “regulation of microtubule‐based process” (GO:0032886), “regulation of microtubule cytoskeleton organization” (GO:0070507), “embryonic digestive tract morphogenesis” (GO:0048557), “regulation of spindle assembly” (GO:0090169), and “mitotic spindle elongation” (GO:0000022; Figure S7). Four KEGG terms were identified as overrepresented (*p* < 0.05) in the 2,309 frontloaded genes: “K03768” PPIB, ppiB (peptidyl‐prolyl cis‐trans isomerase B), “K00461” ALOX5 (arachidonate 5‐lipoxygenase), “K19363” LITAF (lipopolysaccharide‐induced tumour necrosis factor‐alpha factor), and “K18171” CMC1 (COX assembly mitochondrial protein 1).

A third (36.5% or 23 genes) of the biomineralisation‐related genes met the constitutive gene frontloading criteria, that is, a greater expression in the flat habitat compared to the slope regardless of treatment (Figure 5, Table S1). These genes included two carbonic anhydrases (STPCA2, STPCA2‐2), with STPCA2‐2 expression 7‐fold higher than all other frontloaded genes (Figure 5). Interestingly, two bicarbonate (HCO_3_^−^) transporters, sodium bicarbonate cotransporter 3‐like isoform X2 and solute carrier family 4 member gamma (SLC4γ), were frontloaded, the latter of which is known to play a critical role in the provisioning of concentrated HCO_3_^−^ for CaCO_3_ deposition (Tinoco et al. 2023; Zoccola et al. 2015). Also amongst the frontloaded genes were several skeletal organic matrix proteins (SOMPs), including von Willebrand factor D and EGF domain‐containing protein‐like, which is involved in cell–cell and cell–substrate adhesion (Ramos‐Silva et al. 2013). A complete list of the frontloaded biomineralisation‐related genes can be found in Table S1.

### 3.5 Differential Expression of Putative Biomineralisation‐Related Genes

**FIGURE 6:**
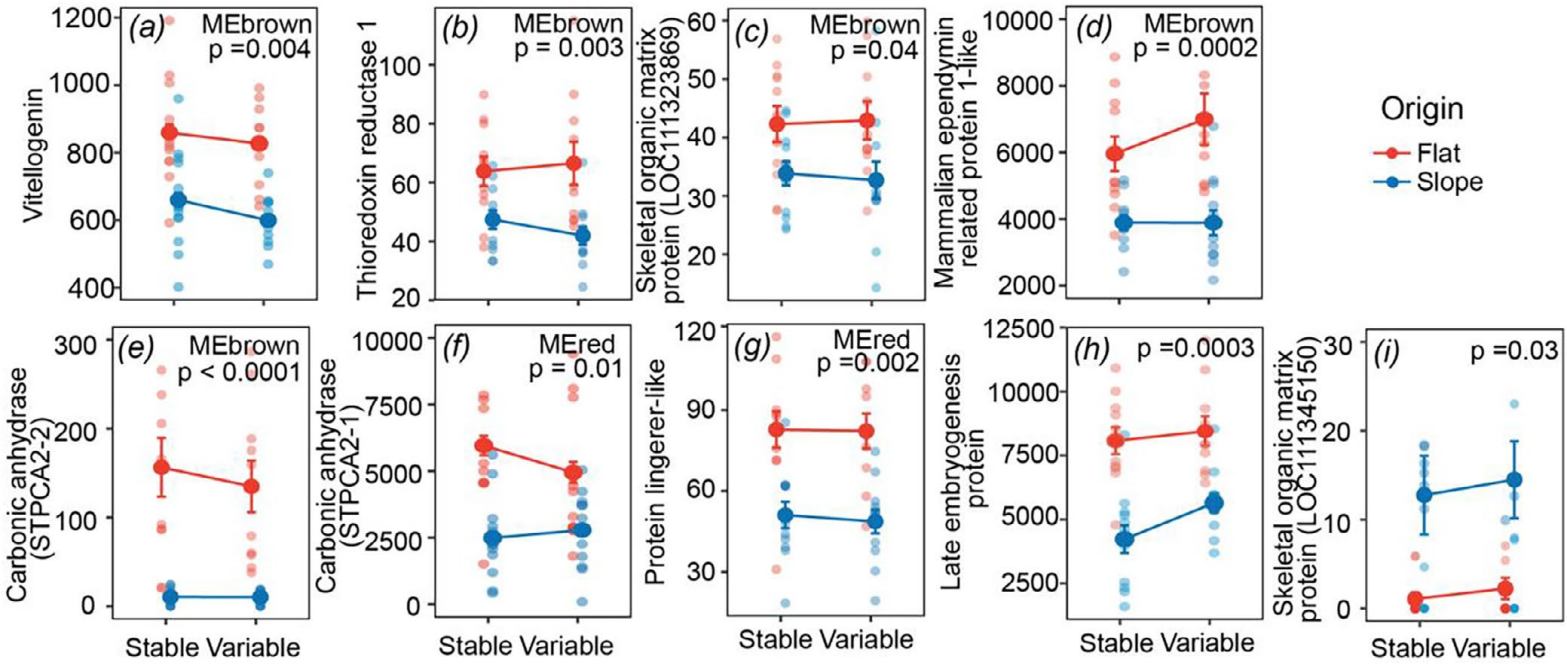
Expression levels of differentially expressed biomineralisation‐related genes. All data are displayed as means ±SE, where points indicate individual measures for coral genets (*n* = 11–12). Statistical significance is displayed for individual effects of origin, as determined from linear mixed effects models. Treatment is indicated on the x‐axis.

A query of the *P. acuta* genome using the 172 putative biomineralisation‐related genes yielded 126 matches. Of these, 64 were present in our dataset (Table S1). None of the 64 biomineralisation genes in our dataset were significantly differentially expressed by treatment or the interaction between treatment and origin; however, 9 of these genes (14%) were significantly differentially expressed by origin. Interestingly, 89% of the differentially expressed biomineralisation‐related genes were upregulated in *P. damicornis* originating from the reef flat relative to the reef slope (Figure 6a–i). Five differentially expressed biomineralisation‐related genes belonged to the MEbrown module (Figure 6a–e) and two belonged to the MEred module (Figure 6f,g)—modules that had significant positive correlation in eigengene expression and net calcification. These genes included carbonic anhydrases (STPCA2 and STPCA2‐2), which are critical in regulating the carbonate chemistry of the calcifying medium where skeletal deposition occurs (Bertucci et al. 2011), and several genes that are related to binding metals such as calcium (thioredoxin reductase 1, mammalian ependymin‐related protein 1‐like, protein lingerer‐like; Peled et al. 2020). The remaining two genes belonged to other WGCNA modules (Figure 6h,i). Only one biomineralisation‐related gene, an uncharacterized skeletal organic matrix protein (LOC1113345150), demonstrated the opposite pattern and was upregulated in *P. damicornis* originating from the reef slope relative to the reef flat (Figure 6j).

## 4 Discussion

### 4.1 Life‐Long Exposure to Extreme pCO_2_ Variability Induces Acclimatory Response in Gene Expression

Biological control of the chemistry at the site of calcification is integral to skeletal formation (Barott et al. 2020; Gilbert et al. 2022; Von Euw et al. 2017) and is particularly important under ocean acidification (Holcomb et al. 2014; Venn et al. 2013). In this study, several genes integral to regulating the chemistry of the extracellular calcifying medium (ECM) were upregulated in *P. damicornis* with a lifelong environmental history of pCO_2_ variability, including carbonic anhydrases and calcium‐binding proteins important to the skeletal ECM. In addition, the expression of skeletal protein vitellogenin, which is understood to contribute to framework building, cell adhesion, and protein–lipid interactions (Mummadisetti, Drake, and Falkowski 2021), was greater in *P. damicornis* originating from the reef flat with extreme diel fluctuations in seawater pCO_2_. Similarly, the expression of skeletal carbonic anhydrase STPCA2‐2, an isoform of STPCA2 (Mummadisetti, Drake, and Falkowski 2021), was up to 14‐fold greater in *P. damicornis* from the reef flat compared to the reef slope. Carbonic anhydrase is critical to intracellular pH (pHi) regulation (Bertucci et al. 2011) and skeletal formation through the conversion of CO_2_ into HCO_3_^−^ (Drake et al. 2013; Moya et al. 2008; Ramos‐Silva et al. 2013), and increasing carbonic anhydrase activity is hypothesised to be a compensatory mechanism of coping with acidification stress (Zoccola et al. 2016). Indeed, our results align with several earlier studies from other natural acidification analogs, which also found carbonic anhydrases upregulated in corals from low pH/high pCO_2_ environments (Kenkel et al. 2018; Leiva, Pérez‐Portela, and Lemer 2023; Radice et al. 2023; Scucchia, Malik, Putnam, et al. 2021; Teixidó et al. 2020). Simultaneously, *P. damicornis* from the reef flat had a robust ability to buffer pHi when exposed to low pH/high pCO_2_ (Brown et al. 2022), suggesting carbonic anhydrase activity may be integral to maintaining acid–base homeostasis of coral populations under ocean acidification. Remarkably, the differential expression of carbonic anhydrase was maintained in *P. damicornis* from the reef flat for 2 months even when exposed to novel, stable pCO_2_ conditions, suggesting constitutive upregulation as opposed to expression plasticity. In fact, constitutive frontloading was identified in > 25% of the dataset, aligning with an earlier study that also demonstrated the constitutive upregulation of stress response genes in corals originally from highly variable environments (Barshis et al. 2013).

The increases in gene expression frontloading in response to high frequency (diel) seawater pCO_2_ variability may stem from co‐tolerance as a result of concurrent exposure to other environmental stressors (e.g., temperature; Vinebrooke et al. 2004). In this study, *P. damicornis* native to the reef flat not only had environmental memory of pCO_2_ variability, but also extreme diel fluctuations in temperature and PAR (Brown et al. 2022)—conditions which may have individually or interactively contributed to coral stress tolerance. While we are unable to disentangle the contribution of each co‐occurring environmental parameter inherent to the habitat of origin, *P. damicornis* native to the stable reef slope subjected to non‐native variable pCO_2_ conditions were able to increase vst‐normalised gene expression over the 2 month experiment (Figure 5 inset), suggesting the significant role of pCO_2_ variability in driving gene expression responses. Priming has been observed in other marine invertebrates under chronic and extreme seawater acidification stress (Gurr et al. 2022). Similarly, our results demonstrate that corals exhibit an acclimatory response in gene expression regulation that may be gained via exposure to short‐term, daily acidification stress over relatively short time scales. Interestingly, recent experimental work within these same reef habitats revealed that *P. damicornis* from the reef flat were also 1°C more heat‐tolerant than conspecifics from the reef slope (Brown, Martynek, and Barott 2024). Together, these results demonstrate that *P. damicornis* displays co‐tolerance to short‐term variability in temperature and pCO_2_. Co‐tolerance has also been observed in other corals, where individuals performing well under one stressor (e.g., seawater acidification, warming, disease) also tended to perform well under every other stressor tested (Wright et al. 2019). This co‐tolerance to multiple stressors is encouraging; however, tolerance to short‐term exposure may not indicate resilience to chronic ocean acidification and warming that will accompany a changing climate, and numerous studies indicate synergistic effects between multiple stressors that decrease coral performance and ecosystem resilience (Anthony et al. 2011; Cornwall et al. 2021; Dove et al. 2020).

### 4.2 Constitutive Frontloading of Coral Biomineralisation Toolkit Promotes Skeletogenesis

Macro‐morphological analyses of net calcification and CaCO_3_ bulk density demonstrated strong effects of origin that aligned with habitat‐specific patterns in water flow and wave exposure (Brown et al. 2022). Specifically, net calcification was significantly greater in *P. damicornis* that originated from the reef flat, whereas CaCO_3_ density was significantly greater in corals that originated from the reef slope (Brown et al. 2022). Skeletal extension, however, did reveal corals from the reef flat increased their extension rates in the stable (non‐native) treatment relative to conspecifics under variable pCO_2_ conditions (Brown et al. 2022). To better resolve changes in biomineralisation resulting from pCO_2_ variability from the effects of co‐occurring physical conditions that exist across environments, micromorphological analysis of *P. damicornis* skeletons were conducted using SEM on areas of new CaCO_3_ deposition that occurred during the 8‐week experiment. This methodology was a powerful way to disentangle pCO_2_ variability, which was not possible to capture with the buoyant weight technique alone. The frontloading of biomineralisation‐related genes in corals with a life‐long environmental history of pCO_2_ variability corresponded with enhanced skeletal formation, aligning with observed changes in primary calcification (i.e., skeletal extension). Nearly half of the biomineralisation‐related genes in our dataset, including carbonic anhydrases, SOMPs (i.e., von Willebrand factor type D domain‐containing protein), and HCO_3_^−^ transporters, were constitutively upregulated in *P. damicornis* native to the reef flat. Notably, the expression of SLC4γ was up to 60% greater in *P. damicornis* from the reef flat compared to the reef slope. SLC4γ supplies HCO_3_^−^ to the site of calcification and therefore is considered one of the most integral genes for skeletogenesis in scleractinian corals (Barott et al. 2015; Tinoco et al. 2023; Zoccola et al. 2015). The supply of HCO_3_^−^ correspondingly increases the pH of the ECM, possibly also contributing to pH regulation (Barott et al. 2015; Zoccola et al. 2015). Interestingly, Zoccola et al. (2015) suggest that SLC4 anion exchangers and carbonic anhydrase (i.e., STPCA2) may interact to form a HCO_3_^−^ transport metabolon to accelerate transmembrane HCO_3_^−^ transport. While this mechanism was not specifically investigated in our study, both isoforms of carbonic anhydrase STPCA2 and STPCA2‐2 were also constitutively upregulated. Accordingly, *P. damicornis* from the reef flat were able to maintain skeletal formation under extreme pCO_2_ variability. Notably, our micromorphological measurements did not include porosity or skeletal density, which are strongly influenced by long‐term exposure (i.e., > 1 year) to low pH in situ (Canesi et al. 2023; Guo et al. 2020; Radice et al. 2023) and require further investigation in response to seawater pCO_2_ variability. Further, the ability to maintain biomineralisation was observed under daily exposure to brief periods of acute acidification stress, leaving open many questions on whether gene expression regulation patterns driven by exposure to environmental variability on a daily basis will continue to encourage biomineralisation under chronic ocean acidification. Nevertheless, long‐term exposure to high‐frequency environmental variability resulted in molecular acclimatisation, whereby corals developed gene expression regulation patterns that enabled them to cope with acute acidification stress.

### 4.3 Increased Photosynthetic Rates as a Mechanism to Cope with Extreme pCO_2_ Variability

Skeletal formation is more energetically demanding under ocean acidification (Holcomb et al. 2014; Ries 2011; Venn et al. 2013). When *P. damicornis* native to the variable reef flat was grown under stable pCO_2_ conditions, an increase in skeletal formation (e.g., the area and number of RADs, coenosteum width) was observed compared to conspecific reef flat natives faced with extreme pCO_2_ variability. This significant increase in biomineralisation when corals were released from stressful pCO_2_ conditions suggests more energy may become available for biomineralisation when, for example, energy is directed away from acid–base homeostasis. Metabolic requirements for scleractinian coral biomineralisation is principally met by the photosynthetic activity of endosymbiotic dinoflagellates (Muscatine 1990). In this study, photosynthetic activity was 20% greater in *P. damicornis* originating from the reef flat (Brown et al. 2022), suggesting an increase in metabolic activity may energetically supplement pHi regulation and biomineralisation on reefs with naturally variable pCO_2_ conditions. Earlier studies from mangrove lagoons (Camp et al. 2019) and CO_2_ seeps (Strahl et al. 2015) have identified increased metabolic activity in several scleractinian species, which may be a common mechanism to cope with the energetic demands of living within extreme environments. In this study, however, differences in metabolic activity were not the result of divergent symbiont communities, with both the reef flat and reef slope populations of *P. damicornis* hosting *Cladocopium latusorum* (Brown et al. 2022). This suggests that the endosymbionts from the reef flat have mechanisms of harvesting more inorganic carbon for photosynthesis, enabling them to overcome the increased energetic demands of biomineralisation under extreme fluctuations in pCO_2_.

## 5 Conclusions

Ocean acidification has led to the thinning of coral skeletons across the world's coral reefs (Guo et al. 2020), and the decreasing strength of the framework of coral reef ecosystems will only accelerate as the climate continues to change (Dove et al. 2020; Eyre et al. 2018). In this study, we identify molecular, cellular, and morphological responses that result in an improved ability to cope with low pH/high pCO_2_ in corals that historically experienced extreme daily fluctuations in pCO_2_ conditions. Lagoonal habitats (i.e., reef flats) make up > 40% of the geomorphological habitats on the Great Barrier Reef (Lyons, Larsen, and Skone 2022) and the resilient corals that inhabit these reefs warrant further investigations as the climate continues to change. As this study found constitutive frontloading of stress‐response genes persisted and biomineralisation increased following transplantation to more stable conditions, corals from these habitats represent ideal candidates for active interventions such as restoration and assisted evolution (van Oppen et al. 2015), particularly as elevated thermal tolerance can also be maintained following transplantation to more stable habitats (Barott et al. 2021; Marhoefer et al. 2021). Given the recognised resilience of corals from highly variable habitats to both elevated temperatures (Barshis et al. 2013; Brown, Martynek, and Barott 2024; Voolstra et al. 2020) and ocean acidification (Brown et al. 2022), these corals warrant special protection and conservation while societies adopt strict policies to cease greenhouse gas emissions.

## Author Contributions

K.T.B. and K.L.B. conceived and designed the study. K.T.B., Z.D., M.P.M., and J.D. carried out the study and collected the data. K.T.B., Z.D., H.M.P., and K.L.B. analysed the data. K.T.B. and Z.D. led the writing of the manuscript, and all authors contributed critically to interpretation and revisions. All authors gave final approval for publication.

## Conflicts of Interest

The authors declare no conflicts of interest.

## Supporting information


Data S1.

## Data Availability

Original data and all R‐scripts generated for this study can be found on BCO‐DMO Project ID 843347 and Zenodo 10.5281/zenodo.14041606.
