# Supplementary material for: Extreme Environmental Variability Induces Frontloading of Coral Biomineralisation Genes to Maintain Calcification Under pCO2 Variability
Source: Mol Ecol. 2024 Nov 28;34(2):e17603. doi: 10.1111/mec.17603 (PMC11701869; doi:10.1111/mec.17603)
Supplement: Supplementary file 1 — Data S1. [file MEC-34-e17603-s001.pdf]

Supplemental Information for:

Extreme environmental variability induces frontloading of coral biomineralization genes to maintain calcification under pCO_2 variability

Kristen T. Brown, Zoe Dellaert, Marcelina P. Martynek, Julia Durian,
Tali Mass, Hollie M. Putnam and Katie L. Barott

Supplementary text

Results

Gene ontology (GO) enrichment of differentially expressed genes by origin was performed by comparing the GO terms from the 840 differentially expressed genes by origin (6,768 terms) to all GO terms in the dataset (17,742 terms). A total of 149 GO terms in the Biological Process (BP) ontology were overrepresented ($p < 0.05$) in the differentially expressed genes. The most overrepresented terms included “regulation of cardiac muscle cell membrane potential” (GO:0086036), “negative regulation of steroid metabolic process” (GO:0045939), “purine deoxyribonucleotide metabolic process” (GO:0009151), “purine deoxyribonucleoside triphosphate metabolic process” (GO:0009215), and “collagen metabolic process” (GO:0032963). A total of 21 KEGG terms were identified as overrepresented in the 840 differentially expressed genes; the most overrepresented included: “lysosomal acid lipase/cholesteryl ester hydrolase” (K01052), “sodium/potassium-transporting ATPase subunit alpha” (K01539), “ATP-binding cassette, subfamily B (MDR/TAP), member 1” (K05658), “transglutaminase 1” (K05619), and “DDB1- and CUL4-associated factor 1” (K11789). No KEGG terms were found significant by adjusted p-value.

Of the 346 differentially expressed genes by origin (representing 4,820 GO terms) that were upregulated in corals from the Slope origin in both treatments, 318 GO terms in the BP ontology were overrepresented ($p < 0.05$).

Of the 481 differentially expressed genes by origin (representing 4,682 GO terms) that were upregulated in corals from the Flat origin in both treatments, 236 GO terms in the BP ontology were overrepresented ($p < 0.05$).

Gene ontology (GO) enrichment of differentially expressed genes by treatment was performed by comparing the GO terms from the 18 differentially expressed genes by treatment (249 terms) to all GO terms in the dataset (17,742 terms). A total of 33 BP GO terms were overrepresented ($p < 0.05$) in the genes differentially expressed by treatment. The most overrepresented terms included “regulation of dopamine uptake involved in synaptic transmission” (GO:0051584; GO:0051586), “regulation of catecholamine uptake involved in synaptic transmission” (GO:0051940; GO:0051944), “snRNA export from nucleus” (GO:0006408), “GDP-L-fucose biosynthetic process” (GO:0042350; GO:0042351), “GDP-L-fucose metabolic process” (GO:0046368), and “snRNA transport” (GO:0051030). A total of 6 KEGG terms were identified as overrepresented in the 18 differentially expressed

genes: “pre-mRNA-splicing factor ISY1” (K12870), “GDP-L-fucose synthase” (K02377), “phosphorylated adapter RNA export protein” (K14291), “endoglucanase” (K01179), “ATP-dependent RNA helicase DDX60” (K20103), and “sulfotransferase” (K01025).

Of the 18 differentially expressed genes by pCO_2 treatment, four were not also significant by habitat of origin. Two of these genes had higher expression in the variable treatment compared to the stable treatment and were annotated as “phosphorylated adapter RNA export protein (PHAX)” and “Sulfotransfer_1 (Sulfotransferase domain).” Conversely, two of the genes (“glycosyl hydrolase family 9”, and unannotated) were higher in the stable compared to the variable treatment. While one gene was not annotated, the other gene was identified as belonging to “glycosyl hydrolase family 9.”

Supplementary Figures

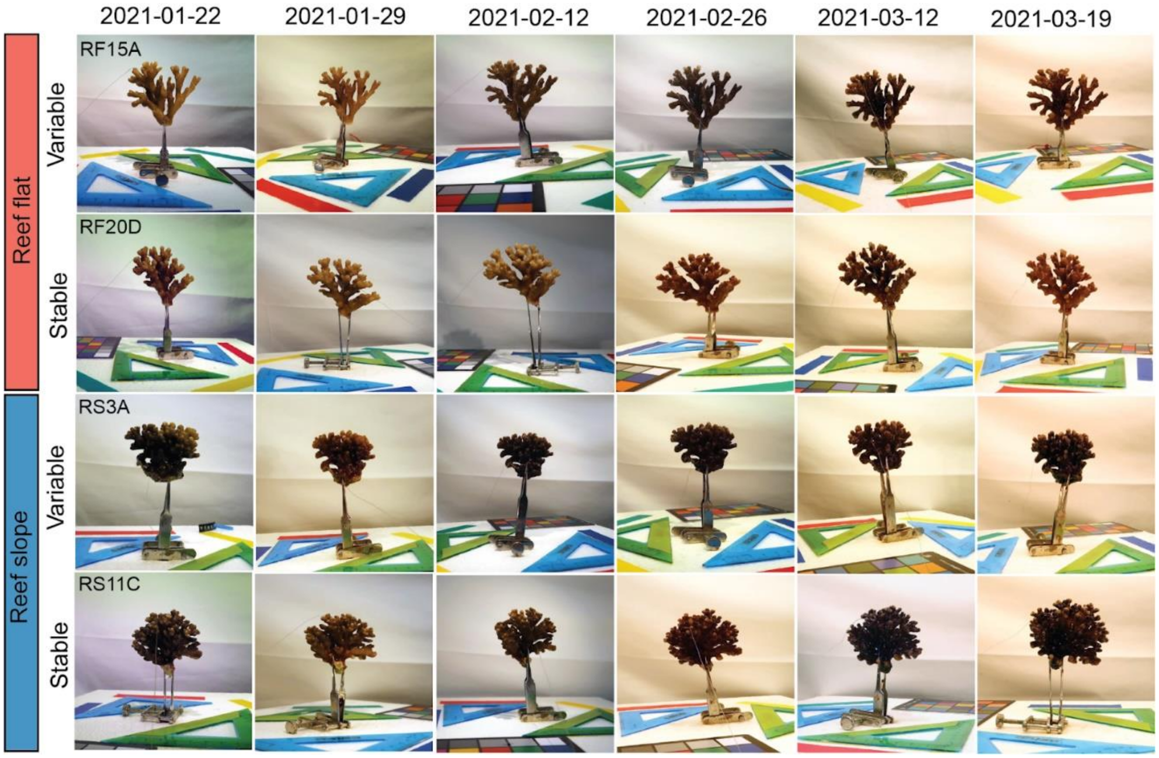

Figure S1. Representative images of *Pocillopora damicornis* fragments over the experiment. Areas of calcium carbonate deposition that occurred during the 8-week experiment were quantified through three-dimensional photogrammetry.

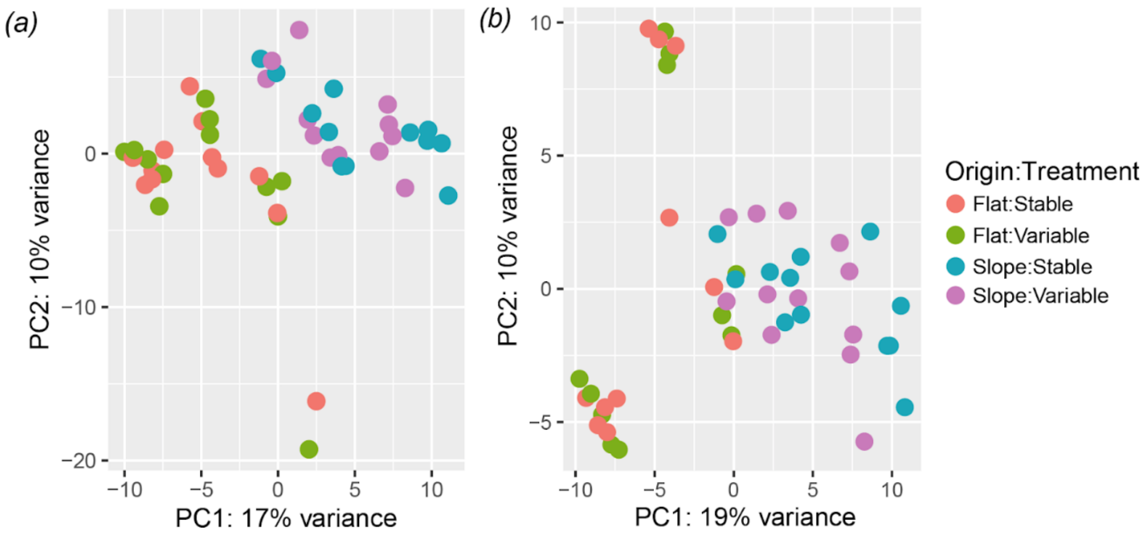

Figure S2. Identification of outliers in the gene expression data. Principal component analysis (PCA) plots of the vst-normalized gene expression matrix for (a) the complete dataset and (b) the dataset with the two outliers removed (two samples of the same genotype, RF16).

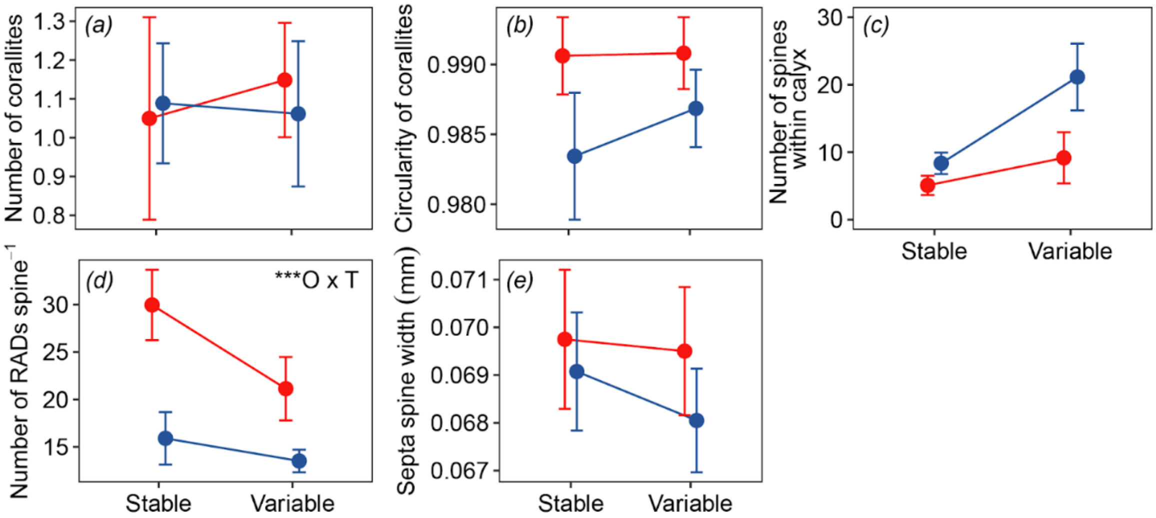

Figure S3. Comparison of micro skeletal morphometrics between origin and treatment. Surface morphology was imaged by scanning electron microscopy (SEM) for features of interest: (a) number of corallites, (b) circularity of corallites, (c) number of spines within calyx, (d) number of rapid accretion deposits (RADs), and (e) septa spine width. Insets indicate statistical significance (***p < 0.0001) of individual and interactive effects for origin (O) and treatment (T) as determined from linear mixed effects models. Reef flat origin, red; reef slope origin, blue. Treatment is indicated on the x-axis.

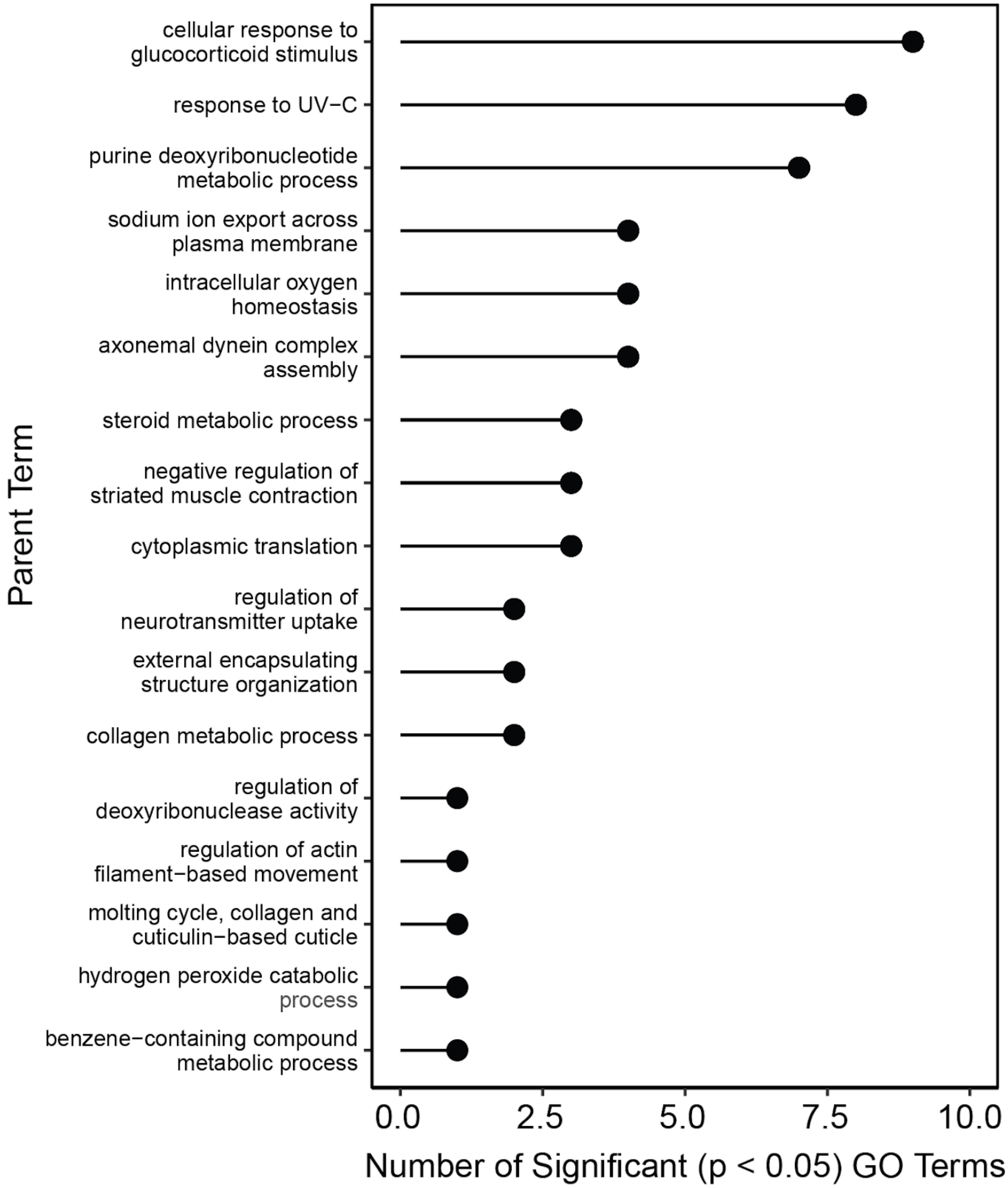

Figure S4. Gene ontology enrichment analysis of differentially expressed genes by Origin. Number of significant ($p < 0.05$) gene ontology (GO) terms are plotted by parent term.

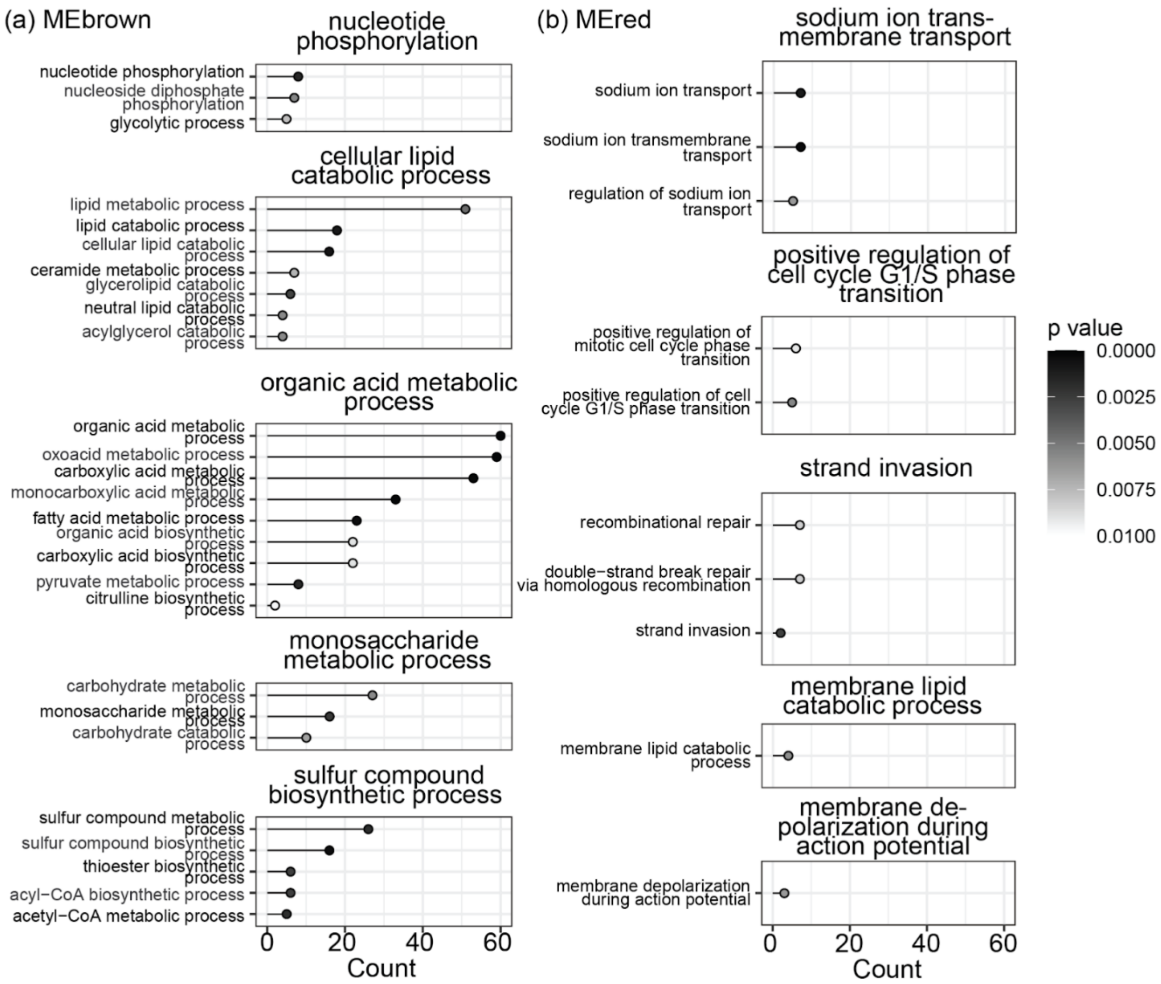

Figure S5. Gene ontology enrichment analysis of WGCNA modules that displayed positive expression in calcification. Number of significant ($p < 0.01$) gene ontology (GO) terms for (a) MEbrown and (b) MEred modules are plotted by parent categories, with the color indicating significance (p value).

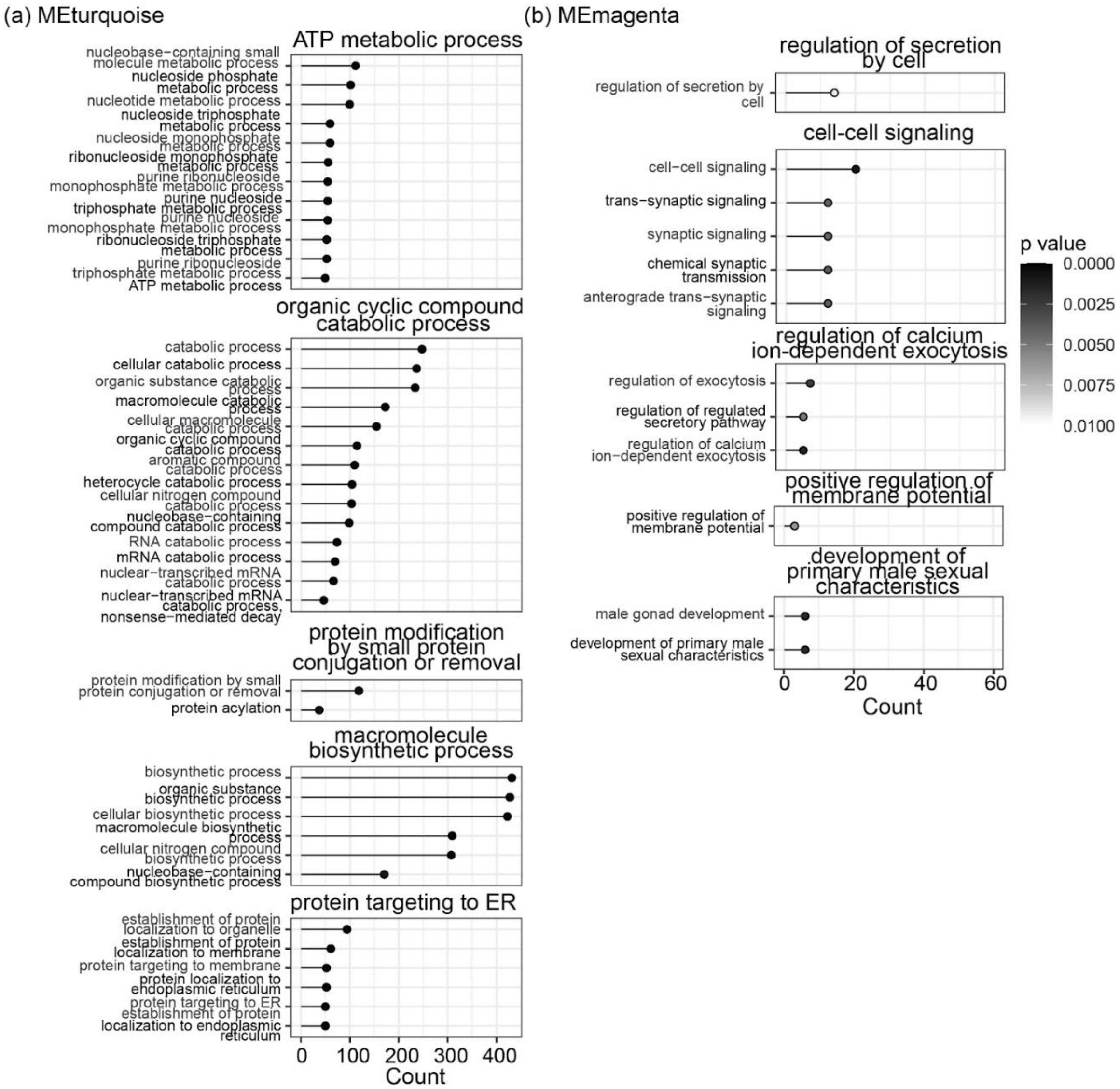

Figure S6. Gene ontology enrichment analysis of WGCNA modules that displayed negative expression in calcification. Number of significant ($p < 0.01$) gene ontology (GO) terms for (a) MEturquoise and (b) MEmagenta modules are plotted by parent categories, with the color indicating significance (p value).

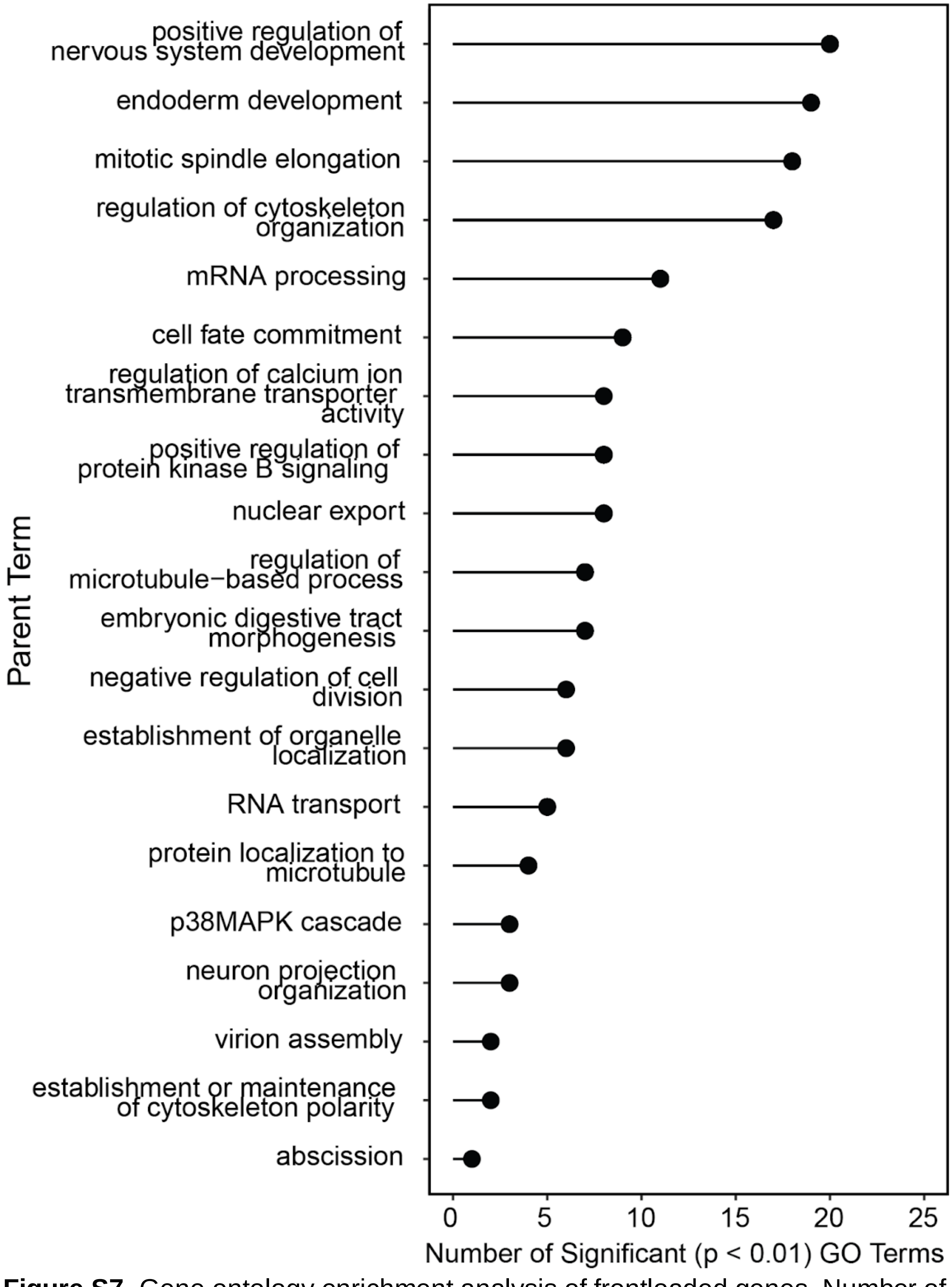

Figure S7. Gene ontology enrichment analysis of frontloaded genes. Number of significant ($p < 0.01$) gene ontology (GO) terms are plotted by parent term.

Supplementary Tables

Table S1. Literature generated biomineralization-related genes (Scucchia, Malik, Putnam, et al., 2021; Scucchia, Malik, Zaslansky, et al., 2021) that met the constitutive gene frontloading criteria (e.g., greater expression in the flat habitat compared to the slope regardless of treatment). 'Pocillopora_actua_best_hit' is the best match of each coral biomineralization-related gene in the genome of *P. acuta* (Stephens et al., 2022).

Pocillopora_acuta_best_hit	Accession number /geneID	Definition	Reference
Pocillopora_acuta_HIv2___RNAseq.g21501.t1	AAD11470.1	L-type calcium channel alpha-1 subunit	Zoccola et al., 1999
Pocillopora_acuta_HIv2___RNAseq.g27976.t1	AAR13013.1	plasma membrane calcium ATPase [Stylophora pistillata]	Zoccola et al., 2004
Pocillopora_acuta_HIv2___TS.g12304.t1	ACE95141.1	carbonic anhydrase [Stylophora pistillata]	Moya et al., 2008
Pocillopora_acuta_HIv2___RNAseq.g16280.t1	AGE35225.2	CARP1 [Stylophora pistillata]	Mass et al., 2013
Pocillopora_acuta_HIv2___RNAseq.g15280.t1	AJQ31790.1	solute carrier family 4 member gamma [Stylophora pistillata]	Zoccola et al., 2015
Pocillopora_acuta_HIv2___RNAseq.g16433.t1	aug_v2a.02830	polycystic kidney disease 1-related (PKD1-related) protein	Takeuchi et al., 2016
Pocillopora_acuta_HIv2___RNAseq.g6446.t1	aug_v2a.05945.t1	TSP-1 and VWA domain-containing	Takeuchi et al., 2016
Pocillopora_acuta_HIv2___RNAseq.g26221.t1	aug_v2a.06122.t1	EGF and laminin G domain-containing protein	Takeuchi et al., 2016

Pocillopora_acuta_HIv2___RNAseq.g25935.t1	aug_v2a.09968.t1	MAM and LDLr domain-containing protein	Takeuchi et al., 2016
Pocillopora_acuta_HIv2___TS.g1545.t1b	aug_v2a.15064.t1	Cystein-rich	Takeuchi et al., 2016
Pocillopora_acuta_HIv2___RNAseq.g27566.t1	aug_v2a.24015.t1	Hephaestin-like protein	Takeuchi et al., 2016
Pocillopora_acuta_HIv2___RNAseq.g13824.t1	Gene:g27814	Annotated: carbonic anhydrase (STPCA2-2)	Mummadisetti et al., 2021
Pocillopora_acuta_HIv2___TS.g23724.t1a	JR997000.1	Uncharacterized skeletal organic matrix protein-3 (USOMP-3)	Ramos-Silva et al., 2013
Pocillopora_acuta_HIv2___TS.g15792.t1	P27_g18472	Integrin - alpha	Drake et al., 2013
Pocillopora_acuta_HIv2___RNAseq.g16715.t1	P28_g11651	Late embryogenesis protein	Drake et al., 2013
Pocillopora_acuta_HIv2___RNAseq.g14653.t1	P8_g9654	Major yolk protein	Drake et al., 2013
Pocillopora_acuta_HIv2___RNAseq.g8821.t1	PFX26597.1	Complement C3 [Stylophora pistillata]	Peled et al., 2020
Pocillopora_acuta_HIv2___RNAseq.g4085.t1	XP_022798902.1	low-density lipoprotein receptor-related protein 8-like [Stylophora pistillata]	Peled et al., 2020
Pocillopora_acuta_HIv2___RNAseq.g7402.t1	XP_022801463.1	sodium bicarbonate cotransporter 3-like isoform X2	Zoccola et al., 2015

Pocillopora_acuta_HIv2___RNAseq.g18103.t1	XP_022803524.1	digestive cysteine proteinase 1-like [Stylophora pistillata]	Peled et al., 2020
Pocillopora_acuta_HIv2___RNAseq.g5807.t1	XP_022803872.1	spore wall protein 2-like isoform X3 [Stylophora pistillata]	Peled et al., 2020
Pocillopora_acuta_HIv2___TS.g11659.t1	XP_022809269.1	microtubule-associated tumor suppressor 1 homolog isoform X1 [Stylophora pistillata]	Peled et al., 2020
Pocillopora_acuta_HIv2___RNAseq.g28226.t2	XP_022810585.1	von Willebrand factor D and EGF domain-containing protein-like, partial [Stylophora pistillata]	Peled et al., 2020
